# PINCER: improved CRISPR/Cas9 screening by efficient cleavage at conserved residues

**DOI:** 10.1093/nar/gkaa645

**Published:** 2020-08-21

**Authors:** Brendan Veeneman, Ying Gao, Joy Grant, David Fruhling, James Ahn, Benedikt Bosbach, Jadwiga Bienkowska, Maximillian Follettie, Kim Arndt, Jeremy Myers, Wenyan Zhong

**Affiliations:** Oncology Research and Development, Pfizer Worldwide Research, Development and Medical, Pearl River, NY 10965, USA; Oncology Research and Development, Pfizer Worldwide Research, Development and Medical, Pearl River, NY 10965, USA; Oncology Research and Development, Pfizer Worldwide Research, Development and Medical, Pearl River, NY 10965, USA; Oncology Research and Development, Pfizer Worldwide Research, Development and Medical, Pearl River, NY 10965, USA; Oncology Research and Development, Pfizer Worldwide Research, Development and Medical, Pearl River, NY 10965, USA; Emerging Sciences & Innovation, Pfizer Worldwide Research, Development and Medical, New York, NY 10016, USA; Oncology Research and Development, Pfizer Worldwide Research, Development and Medical, San Diego, CA 92121, USA; Oncology Research and Development, Pfizer Worldwide Research, Development and Medical, Pearl River, NY 10965, USA; Oncology Research and Development, Pfizer Worldwide Research, Development and Medical, Pearl River, NY 10965, USA; Oncology Research and Development, Pfizer Worldwide Research, Development and Medical, Pearl River, NY 10965, USA; Oncology Research and Development, Pfizer Worldwide Research, Development and Medical, Pearl River, NY 10965, USA

## Abstract

CRISPR/Cas9 functional genomic screens have emerged as essential tools in drug target discovery. However, the sensitivity of available genome-wide CRISPR libraries is impaired by guides which inefficiently abrogate gene function. While Cas9 cleavage efficiency optimization and essential domain targeting have been developed as independent guide design rationales, no library has yet combined these into a single cohesive strategy to knock out gene function. Here, in a massive reanalysis of CRISPR tiling data using the most comprehensive feature database assembled, we determine which features of guides and their targets best predict activity and how to best combine them into a single guide design algorithm. We present the ProteIN ConsERvation (PINCER) genome-wide CRISPR library, which for the first time combines enzymatic efficiency optimization with conserved length protein region targeting, and also incorporates domains, coding sequence position, U6 termination (TTT), restriction sites, polymorphisms and specificity. Finally, we demonstrate superior performance of the PINCER library compared to alternative genome-wide CRISPR libraries in head-to-head validation. PINCER is available for individual gene knockout and genome-wide screening for both the human and mouse genomes.

## INTRODUCTION

The development of CRISPR/Cas9 represents a significant advancement in genome editing (1–3). Among many applications, *Streptococcus pyogenes* Cas9 empowers functional genomics screens with improved efficacy and specificity over previous RNA interference-based technologies (4–7). In these screens, single-guide RNAs (‘sgRNAs’ or ‘guides’) direct Cas9 to induce mutations in one gene per cell across many genes in a population of many cells. Next, a selection pressure is applied to the population for a cellular phenotype such as impaired proliferation. Finally, guides are sequenced and counted, and the cellular dependence on each gene is assessed by population-level changes in guide abundance. CRISPR/Cas9 screens are particularly exciting platforms for oncology target discovery because they can uncover unique dependencies of oncogene addiction, lineage-specific regulators, synthetic lethal vulnerabilities of drug treatment, vulnerabilities of tumor immune evasion in genetically engineered mouse models, and physiologically relevant targets of the tumor micro-environment *in vivo* (5,8–11). Because screening sensitivity depends on robust and precise abrogation of gene function, optimizing CRISPR/Cas9 screening is an important methodological effort.

Typically, each gene has hundreds of possible Cas9 targets, controlled by GG dinucleotides which recruit Cas9’s protospacer-adjacent motif (PAM)-recognition domain. While replicate guides targeting multiple positions in the same gene are possible, the practical space requirements of cell culture in genome-wide screening constrain replicate count and most libraries include only four to six sgRNAs per gene. Therefore, it is imperative that guides be as active and specific as possible, and many algorithms exist to predict guide activity from their features, select the most active guides and combine them into screening libraries. In essence, the goal of CRISPR guide design is to destroy the normal function of every gene as effectively as possible using available scores.

Many algorithms use guide sequence to predict experimental Cas9 editing machinery behavior, and we refer to these collectively as sequence features. The first guide selection criterion developed was predicted specificity, and methods evolved from counting off-target alignments to trained off-target scoring models which consider the position and identity of each possible mismatched sgRNA nucleotide (e.g. Hsu *et al.* (12), CFD and Elevation; see Supplementary Tables S1–4 for full reviews of literature, libraries, design tools and scoring algorithms) (12–14). In this manuscript, we use the term ‘specificity’ to mean ‘the predicted absence of sgRNA-attributed Cas9-induced cleavage at sites other than the intended target.’ It was then observed that sgRNA sequences confer variable Cas9 enzymatic cleavage efficiency, hypothetically due to variability in sgRNA-DNA binding energy and/or steric interactions with the Cas9 enzyme. Scoring algorithms were developed to predict cleavage efficiency from sgRNA sequence context (so-called ‘on-target’ predictions such as Doench *et al.*’s ‘Rule Set 1’) (15). Similarly, specific sgRNA sub-sequences have been shown to predict high or low sgRNA activity and were proposed as selection criteria (16,17). Cleavage efficacy scores were next improved by thermodynamic modeling of the step-wise association of Cas9:sgRNA with DNA, currently thought to be Cas9:PAM recognition, DNA:DNA unpairing, base pairing of PAM-proximal sgRNA ‘seed’ nucleotides to DNA and finally pairing of PAM-distal sgRNA to DNA (e.g. Doench *et al.*’s ‘Rule Set 2’ and CRISPRater) (13,18–20). Most recently, cleavage efficacy scores have been further improved by use of either more sophisticated machine learning approaches using more training data (e.g. DeepCas9, TUSCAN, DeepSpCas9), or better *a priori* models of Cas9 cleavage thermodynamics with the added benefit of generalizing to off-target cleavage (e.g. uCRISPR, CRISPRspec) (21–25). In this manuscript, we use the term ‘cleavage efficacy’ to mean ‘the predicted extent of sgRNA-attributed Cas9-induced enzymatic cleavage at the intended target,’ and later as an alternate descriptor for ‘Broad Rule Set 2 score.’ Some sgRNA sequences have been shown to cause unintended folding of the larger sgRNA scaffold and proposed as exclusion criteria (e.g. CHOPCHOPv3) (26,27). An orthogonal consideration is that most sgRNA expression vectors use the U6 promoter to drive RNA Pol III transcription of the sgRNA, but Pol III transcription is terminated via catalytic inactivation and backtracking by contiguous runs of 1–6 thymine nucleotides (RNA: uracils), with described critical cutoffs in human cells of length four (by experimental validation, and used by most design algorithms which consider polyT), or length three (in screening) (19,28–29). Finally, it was discovered that the sequence context of Cas9 cut sites affect microhomology-mediated end joining repair, so algorithms were developed to predict mutational consequences of each Cas9 target and prioritize targets expected to maximize the percentage of frameshifting edits in the target gene (30–33). However, Doench *et al.* reported that early microhomology prediction rules did not improve upon efficiency prediction, He *et al.* reported that only 11.2% of sgRNAs in a large training dataset had predicted in-frame probabilities >0.5, and newer algorithms describe misrepair as being highly dependent on cell line and apparently also cell lineage as the 1 nt indels modeled by predictive algorithms contrast sharply with descriptions of insertions longer than 25 nt in primary T cells (>90% of edits), and multi-kilobase deletions and complex rearrangements in embryonic stem cells (13,30–36). At least 15 genome-wide human CRISPR libraries have been developed which incorporate varying guide-intrinsic features and implementations (e.g. Avana, Brunello, TKOv3. See Supplementary Table S2) (13,15,37).

Beyond intrinsic features of guide sequences, several features of guide targets have also been proposed as selection criteria. First, it was discovered that Cas9 editing can affect exon inclusion during RNA splicing by abrogating splicing factor recognition motifs (so-called exonic splicing enhancers or ‘ESEs’), and it was proposed that targeting these motifs could knock out exons with lengths that are not a multiple of 3 nt (‘asymmetric exons’), thus frameshifting the mature mRNA to drive gene knockout via nonsense-mediated decay (NMD) (38,39). Similarly, it is possible to assess the percentage of edits at any position in the transcript which will induce NMD by the ‘50-nt rule,’ which dictates that premature stop codons positioned greater than 50–55 nt from the most 3’ exon junction complex induce NMD (e.g. CRISPRO), and others have approximated this approach by avoiding the 3’ end of the coding sequence (CDS, e.g. the Whitehead library) (5,40–41). Alternatively, among other approaches to isoforms some groups have suggested targeting expressed exons (e.g. GUIDES) (42). Polymorphisms and somatic variants can both impair sgRNA:DNA binding at the target and enhance unexpected off-target sgRNA:DNA binding (e.g. CRISPR Off-Target Tool) (43). Adding another dimension is the discovery that regions of open chromatin are more amenable to Cas9 editing than regions of closed chromatin, which was shown to be caused by nucleosomes blocking Cas9 from accessing PAMs (19,44). Some design tools account for this by considering DNAse hypersensitivity at the target (e.g.CRISPR-DO) or off-targets (e.g. CROP-IT), although others have shown chromatin accessibility poorly predicts guide activity (DeepSpCas9) (23,45–46). Next, Shi *et al.* showed that targeting Cas9 to essential domains dramatically improves functional inactivation of target genes, presumably by affecting gene function even with in-frame edits, and some guide design tools incorporated domains as a guide selection criterion (e.g. PAVOOC) (47,48). However, domain annotations are often sparse for poorly characterized gene families and short structural proteins can be entirely annotated as single domains. To solve the sparsity of domain annotations and increase resolution, other groups have described targeting highly conserved nucleotides (CRISPR-DO, CRISPR-FOCUS), amino acids by substitutions (CROATAN, Protiler), or amino acids by deletions (CRISPRO) to improve sgRNA activity (36,41,45,49–50). The rationale that gene regions unaltered through evolutionary history are under purifying selection and are therefore likely to be essential for gene function is a classic and effective framework in comparative genomics (51). While some regions of protein sequence are relatively tolerant of minor alterations, the identification of coding space that has maintained strong evolutionary conservation identifies highly constrained protein structural elements that are likely functionally intolerant of in-frame indels caused by CRISPR/Cas9. In this manuscript, we use the terms ‘guide activity’ and ‘guide effectiveness’ to mean ‘the extent of sgRNA-attributed abrogation of target gene function’.

Critically, few guide design tools and only one genome-wide library consider optimization of both guides themselves (i.e. enzymatic activity) and guide targets (i.e. conservation) to maximize phenotypic knockout of target genes (36,41–42,45,48–50). Only four guide design tools provide both enzymatic efficiency predictions and either nucleotide conservation scores (CRISPR-DO, CRISPR-FOCUS) or domain annotations (GUIDES, PAVOOC) (42,45,48–49). However, in all four the weights of these features in guide selection are neither justified by training data nor suggested, and users must weigh features for themselves. The three tools that have come closest to integrating guide and target features are ProTiler, CRISPRO and CROATAN (36,41,50). ProTiler presents a sophisticated method to predict the most CRISPR-sensitive regions of genes (including novel domains), by incorporating substitution-based protein conservation (SIFT), percentage of transcripts covered, amino acid identity and annotations of domains (Pfam), protein secondary structure and post-translational modifications into a gene targeting score, but critically omits enzymatic efficiency prediction and does not present a guide library (36). CRISPRO presents a guide scoring method which incorporates deletion-based protein conservation (PROVEAN), protein secondary structure aggregated from four tools (PSSpred, PSIpred, SPINE X and RaptorX), domains (Interpro), exon symmetry, strand, amino acid identity and disorder, NMD, guide specificity (Hsu *et al.* (13)), GC content and dinucleotide positional identities (similar to first-generation enzymatic on-target scores) (41). While CRISPRO is critically supported by adequate training data, it omits second-generation enzymatic efficiency prediction (e.g. Doench *et al.* (13)) and presents no guide library since it is intended as a screen reanalysis tool. Finally, among the 15 genome-wide CRISPR libraries we reviewed, no libraries considered domains, and the only library to consider conservation was CRoatan (Supplementary Table S2) (50). CRoatan considers substitution-based protein conservation (PROVEAN), misrepair prediction (Bae *et al.* (30)), polyT (≥4), and a cleavage efficacy score re-fit using data from Doench *et al.* and Chari *et al.*, but critically omits consideration of guide specificity scoring (aiming instead to glean specificity from dual-guide targeting), second-generation enzymatic efficiency prediction (e.g. Doench *et al.*), and domains (15,50,52).

We hypothesized that joint optimization of Cas9 enzymatic activity and gene feature targeting would yield better guides than either approach alone. Further, we hypothesized that deletion-based protein conservation would best simulate in-frame Cas9 edits and outperform other gene targeting methods such as domains, nucleotide conservation, and substitution-based protein conservation. A significant fraction of Cas9-induced edits likely leave the reading frame intact (missense mutations, in-frame indels), and a method which ensures those edits affect critical gene regions will likely achieve better gene knockout. Finally, we aimed to use a data-driven approach to clarify which of the overwhelming array of published sgRNA features best predict activity and how best to combine them.

Here, we built the most comprehensive CRISPR/Cas9 sgRNA feature database to-date for both mouse and human genomes, including a novel genome-wide conservation score based on single amino acid deletions and novel mapping of protein domains to genomic coordinates. We aggregated the largest yet assembled training dataset of CRISPR-tiled essential genes to test the predictive value of guide and target features (and their combinations) and discovered not only that enzymatic efficiency and protein conservation are the best predictors of guide activity, but that their effects are additive. We also observe that guides containing polyT homopolymers as short as three consecutive thymidines (TTT) exhibit reduced activity, which contrasts with the prevailing cutoff in the field (TTTT) and suggests further modification to existing sgRNA scaffolds (e.g. F+E, LRG2.1) for improved RNA Pol III transcription (53,54). Next, we trained an algorithm to combine effective features (specificity, enzymatic efficiency, conservation, domains, homopolymers, CDS position, restriction sites, polymorphisms) and present a novel genome-wide CRISPR library which for the first time incorporates deletion-based ProteIN ConsERvation (the PINCER library). We constructed PINCER using an optimized sgRNA scaffold (LRG2.1), and while many libraries present only a human version we also present an equivalent mouse genome-wide library to enable *in vivo* pre-clinical drug target validation and immune-oncology target discovery (54,55). Finally, we prove in direct head-to-head validation that PINCER guides outperform Avana and TKOv3 in terms of guide activity, and that PINCER guides outperform Avana, Brunello and TKOv3 in terms of intra-gene guide concordance. The PINCER human and mouse genome-wide libraries are included with this manuscript (Supplementary Tables S22 and 23), and our complete genome-wide databases are available on GitHub (https://github.com/veeneman/PINCER).

## MATERIALS AND METHODS

### Construction of a CRISPR/Cas9 sgRNA feature database

We extracted all NGG-associated guide sequences and coordinates from the human and mouse reference genomes (hg38, mm10), including unplaced contigs but excluding alternate haplotypes (56). Although early reports suggested that *S. pyogenes* Cas9 also recruits to NAG PAMs, more recent work has shown Cas9’s affinity is much stronger to NGG than all other PAM sequences, so we only considered NGG sgRNAs (13,57).

### Annotation of intrinsic guide features

We scored guide cleavage efficacy using the Microsoft Azimuth 2.0 implementation of the Broad Rule Set 2 score (Python v2.7.13, Numpy v1.12.1, training data V3_model_nopos.pickle) (13). Next, we aligned each guide back to the genome with BatMis v3.00 (a Burrows–Wheeler transform aligner similar to BWA or Bowtie but with more detailed alignment reporting), requiring the ‘GG’ PAM dinucleotide and allowing up to 5000 alignments with up to four mismatches and aggregated these into a single specificity score using our own implementation of (Hsu *et al.*) (12,58). We repeated this process using alignments to known protein-coding sequence to generate a proteome-wide specificity score, using Gencode-basic genes with transcript support level 1 (human v27, mouse vM15) (59). We tracked whether guides exceeded 5000 alignments, and the number of alignments with 0–4 mismatches. Considering complete sgRNA scaffolds (Supplementary Table S19), we also tracked maximum homopolymer lengths in each guide (polyA, polyC, polyG, polyT) and whether guides contained inadvertent Esp3I restriction sites (CGTCTC). Finally, we predicted each guide's melting temperature using the Bioconductor HELP library's implementation of (Allawi and SantaLucia), which in our hands was much faster than the popular Primer3 tool and while offset by about 10°C produced nearly identical rank-ordered results (Supplementary Figure S4) (60–62).

### Annotation of guide target features

In order to annotate features of guide targets we mapped feature tracks to the genome and intersected them with guide cut sites. We started by annotating principal isoforms (APPRIS, v108 human, v106 mouse) of Refseq protein-coding genes (v109) (63,64). We tracked and considered targeting consensus protein sequence across isoforms, but favored principal isoforms after observing that low-confidence short isoforms hinder domain targeting (data not shown). Next, we tracked each guide's position in the target's coding sequence (%CDS, alternatively referred to as 3’%), the percentage of frameshifts capable of inducing NMD by our own implementation of ‘the 50-nt rule,’ the ‘symmetry’ of each exon (whether removal of the exon will result in frame-shifting of downstream exons), and via amino-acid coordinates, conserved domain database (CDD) regions (typically domains—human: accessed 16 February 2018, mouse: accessed 23 February 2018), CDD sites (typically catalytic residues, interaction sites, etc.) and Uniprot secondary structure (alpha helices, beta sheets, turns and coiled-coil regions, v2017_09) (40,65–66). We annotated exonic splicing enhancers (‘ESEs’) by searching for 84 known enhancer sequences within principal isoform exons (the ‘INT3’ set) (67). Finally, we appended genome-anchored information including Pfam domains (generated using University of California, Santa Cruz (UCSC) genes, v2017–04-07), and common polymorphisms expected to disrupt any position in the 23 nt guide+PAM (≥10% variant allele frequency (VAF) in dbSNP v147, human only) (68–70). In this manuscript we use ‘domain’ to mean ‘a region of amino acids in a protein, identified by ortholog comparisons and potentially annotated with known or predicted function’ (e.g. CDD, PFam), ‘protein secondary structure’ to mean ‘a region of amino acids in a protein known or predicted to form a local biochemical structure’ (e.g. Uniprot alpha helices) and ‘protein conservation’ to mean ‘an amino-acid-resolution score which tracks invariability across known orthologs’ (e.g. our novel conservation score).

### Generation of a novel genome-wide conservation score

We downloaded data from a published experiment in which the homology-dependent repair (HDR) competency of a series of BRCA1 RING domain mutations was experimentally tested (71). These data included mutation effect predictions from public tools (SIFT, Polyphen, CADD and GERP), and a specific prediction combining experimentally measured binding affinity of BRCA1 to its heterodimer BARD1 and E3 ligase activity of the BRCA1–BARD1 complex (‘HDR_predictions’). We then predicted mutation effects using PROVEAN, a conservation-based variant effect predictor which compares alignment of the wild type protein to an ortholog set versus alignment of the mutant protein to the same ortholog set (72). We included predictions using PROVEAN’s web app, and using the command-line tool with either the 2011 BLAST non-redundant database (used in the PROVEAN publication), or the current 2017 version (Supplementary Table S7) (73).

Next, we compared configurations of PROVEAN to nucleotide conservation metrics and their correlation with phenotypic readout of sgRNA activity. First, we aggregated public sgRNA tiling data for the essential gene PLK1 (from Munoz *et al.*, average of three cell lines), the MAPK-pathway-inhibitor resistance gene MED12 (from Doench *et al.*, average of two inhibitors) and the contextually essential mouse gene Smarca4 (from Shi *et al.*) (see section ‘Model Training Dataset’ for more details) (13,47,74). Next, we ran PROVEAN while modulating three parameters in 2 × 2 × 3 = 12 configurations. These parameters were: single-amino-acid deletions versus substitutions (averaging all 20), the position-weight matrix BLOSUM62 (default) versus a custom identity matrix (scored: +5 for identical AAs and −2 for different) and three ortholog sets: the 2011 BLAST non-redundant (‘NR’) database (used in the original PROVEAN publication), the 2011 BLAST non-redundant database with low-evolutionary-distance proteins ‘pruned’ (‘NRp,’ specifically removing synthetic, Human, Orangutan, Macaque and Chimpanzee proteins), or manually curated ortholog sets from Homologene (Supplementary Table S8) (73,75). Next, we used the UCSC table browser to download the nucleotide conservation tracks phyloP, phastCons and phastConsElements for human (hg38, 100way alignments) and mouse (mm10, 60way alignments), downloaded multiple sequence alignments for the manually curated ortholog sets from Homologene and computed percent identity at each reference residue, and computed moving averages for phyloP (3, 6, 9 nt windows), phastCons (3, 6, 9 nt), and homologene (0–4aa) (Supplementary Table S9) (76).

Next, we compared simulated deletions of varying lengths using PROVEAN. Protein sequences for PLK1, MED12 and Smarca4 were acquired from NCBI, the effect of the deletion of every amino acid (1AA del), every pair of amino acids (2AA del) and every trio of amino acids (3AA del) was assessed using the PROVEAN web app, and multiplied by −1 such that larger positive numbers correspond to higher conservation (Supplementary Figure S23 and Table S26).

Finally, to generate a genome-wide protein conservation score, we used PROVEAN and Blast NR v2011 to predict the detrimental impact of the deletion of every single amino acid in APPRIS principal isoforms of Refseq genes (human and mouse) and multiplied by −1 such that larger positive numbers correspond to higher conservation. We named this score AADelCons, for amino acid deletion conservation.

### Model training dataset

We assembled a comprehesive sgRNA activity training dataset, spanning seven individual datasets of sgRNA activity measured by log2-fold-changes (LFCs) from five publications, in which genes with clear experimental readouts were tiled by sgRNAs in an unbiased fashion. For datasets where increased sgRNA activity was measured as enrichment (either by drug resistance, or by flow-sorting marker-negative populations), we inverted these into negative LFCs ((-)LFCs) as indicated for internal consistency of the training dataset. Likewise, for datasets including sgRNAs targeting genes which were not expected or observed to show activity (e.g. negative controls), we filtered to genes either known to be essential (Achilles 17Q4) or achieving a mean-sgRNA LFC <−1 in all cell lines tested and list exclusions below (8). The sgRNA datasets were: (i) (-)LFCs in flow-sorted antigen-negative cells for cell-surface human genes (15); (ii) (-)LFCs in flow-sorted antigen-negative cells for cell-surface mouse genes (excluding Cd2, Cd3e and Cd53, which the original authors excluded, H2-K which was not targeted specifically and sgRNAs at near-zero abundance in controls) (15); (iii) (-)LFCs in inhibitor-resistant cells in three pooled synthetic lethality proliferation screens (excluding TOP2A, CDK6, MLH1, MSH2, MSH6 and PMS2, which the original authors excluded, CLDN10 which the original authors do not describe and shows no activity with any drug and sgRNAs the original authors QC-flagged) (13); (iv) (-)LFCs in inhibitor-resistant cells in a pooled synthetic lethality proliferation screen (excluding non-exonic sgRNAs) (77); (v) LFCs in a pooled proliferation screen (filtered for essential genes, and excluding non-exonic sgRNAs) (77); (vi) LFCs in a pooled proliferation screen (filtered for essential genes) (74); (vii) LFCs in a pooled proliferation screen (47); see Supplementary Table S10 for a full gene list. We further filtered these input sgRNA<>LFC measurements (*n* = 43 307) to sgRNAs targeting coding sequence in our database (*n* = 41 611), converted LFCs to *z*-scores on a per experiment and per gene basis (see Supplementary Tables S11 and 12 for feature descriptions and data) and filtered to sgRNAs which target specifically (specificity score > 0.50 and zero off-targets in the genome with ≤1 mismatch; *n* = 27 508).

### Gene set definitions for validation experiment

In order to compare the activity of multiple CRISPR libraries across three cell lines (described later), we defined lists of genes expected to be essential to all lines (‘essential genes’), essential to one or two lines (‘cell line-selective genes’), or essential to no lines (‘negative control genes’).

For essential genes, we intersected lists identified by RNA interference (the ‘CCE’ set from Hart *et al.*, *n* = 217) and CRISPR (the ‘CEG’ set from Hart *et al.*, *n* = 684), reasoning that multiple evidence types would yield a robust and conservative essential gene set (*n* = 133) (37,78). We observed good agreement between the two lists, which bolstered our confidence in using the intersection between the two lists and the cutoff of −log10(p) > 3 for our validation experiment (Supplementary Figure S13). We labeled 33 of these for which we had prior screening experience suggesting their strong essentiality, 30 of which were significant in Hart *et al.* (37)’s Bayes factor essentiality scores (*P* < 0.001 in a *t*-test versus 0). Next, noting a discrepancy between apparent essential structural proteins versus enzymes, we annotated the domain architecture of each gene. We defined ‘broad domain’ genes as having a single domain covering >90% of the protein sequence (representing 12% of genes in the genome), and ‘narrow domain’ genes as having either multiple domains covering <85% of the protein sequence, or a single domain covering <50% of the protein sequence (representing 52% of genes in the genome). Finally, we ranked genes by our known significant essential list and the mean Hart ’17 Bayes factor, and selected the top 18 in each domain group for this experiment (see Supplementary Table S14 for scores related to this process).

For cell line-selective genes, we downloaded and joined CRISPR and copy number variation (CNV) data from Achilles (18Q3, *n* = 17 520 genes) for DLD1, HS766T and HCC1428. We identified pairwise differential vulnerabilities by a CERES score difference greater than 0.5 and with copy-neutrality in both lines (2.5 > copy number > 1.5) (*n* = 177 total genes). Of these, we automatically included all kinases (*n* = 5) and transcription factors (*n* = 9) in the experiment. Using the remaining 163 genes, we identified the top two differential vulnerabilities in both directions for each line versus the other two as: CERES difference >0.50 for each cell line versus cell line comparison, CERES <−0.50 in sensitive lines, CERES > −0.30 in non-sensitive lines (except in one case which didn’t yield two genes) and taking the top two based on mean CERES difference (*n* = 12) (see Supplementary Table S15 for scores related to this process).

For negative control genes, we used the Achilles data (18Q3) to identify copy-neutral genes in all three cell lines with low mean CERES scores (|CERES| < 0.2) and low standard deviations across cell lines (sd < 0.15). Of these, we arbitrarily selected fifteen solute carriers (SLCs) and 13 olfactory receptors (ORs) (see Supplementary Table S16 for scores related to this process).

### Plasmid construction and sgRNA cloning

To construct sgRNA expression vectors, 83-nt DNA sequences were assembled by flanking 20nt guide protospacers (Supplementary Table S19) with Esp3I restriction sites and polymerase chain reaction (PCR) primer templates (Supplementary Table S20). Lyophilized and cleaved 83mers were ordered from Agilent (5000 oligo ‘SureGuide Unamplified Custom CRISPR Library’, Part Number: G7555B), re-suspended in water and amplified by PCR. Next, guides were excised by Esp3I at 21°C for 3 h and ligated into a modified version of Tarumoto *et al.*’s LRG2.1 vector, in which green fluorescence protein was replaced by puromycin resistance (*pac*) using BamHI+BsrGI (54,55). Finally, vectors were electroporated into four replicates of STBL4 cells and pooled.

A constitutive Cas9 expression vector was constructed as follows. First, an empty lentiviral vector with ampicillin resistance (pLV7-Empty) was constructed by restricting pLenti7.3/V5-DEST (Thermo Fisher Scientific, #V53406) with ClaI and Acc65I, then re-ligating by duplexed oligonucleotides (IDT, Supplementary Table S19). The EF-1 Alpha short (EFS) promoter was added (pLV7-EFS), by PCR amplifying EFS, restricting pLV7-Empty with XbaI and BamHI, and ligation. Next, the reverse-complement DNA sequence encoding human-optimized 3× FLAG-tagged Cas9 was extracted from its source publication (79). Cas9 DNA was synthesized in three fragments with flanking BamHI and NheI restriction sites (ATUM, DNA2.0), 2A self-cleaving peptide sequence (P2A) oligonucleotides were synthesized (IDT, Supplementary Table S19) and the hygromycin resistance gene (*hph*, Hygro) was synthesized with flanking Esp3I and MluI restriction sites (ATUM, DNA2.0). Finally, Cas9, P2A and Hygro were ligated together, pLV7-EFS was restricted by BamHI and MluI, and Cas9-P2A-Hygro was inserted by ligation to generate the final vector (pLV7-EFS-Cas9-P2A-Hygro). sgRNA and Cas9 cloning vectors were illustrated using AddGene's Giraffe software, https://github.com/addgene/giraffe (Supplementary Figure S16) (80).

### Cell culture

Cell lines were purchased from the American Type Culture Collection (ATCC). HEK293T, DLD-1 and HCC1428 were maintained in Dulbecco's modified Eagle's medium (DMEM) + 10% fetal bovine serum (FBS) and Hs776T was maintained in Roswell Park Memorial Institute media (RPMI) + 10% FBS, with HEK293T also receiving high glucose, pyruvate and 1% penicillin/streptomycin (Thermo Fisher Scientific), and HCC1428 also receiving 1% HEPES. All cells were maintained in humidified incubators at 37°C and 5% CO_2_. Cell lines stably expressing Cas9 were generated by lentiviral transduction at a low multiplicity of infection (MOI) to introduce a single Cas9 copy per cell and selected using hygromycin at 250 μg/ml (DLD1) or 400 μg/ml (Hs766T and HCC1428). Cells expressing sgRNA were selected using puromycin at 2 μg/ml.

### Lentiviral transduction

Lentivirus was produced by transfecting cells with helper plasmids. Briefly, 10 mg of plasmid DNA, 5 mg of VSVG, 7.5 mg of psPAX2 and 40 mL of 1 mg/ml PEI were mixed, incubated and added to the 10 cm plate HEK293T cells. Media was replaced 6–8 h post-transfection. Virus was collected 48 and 72 h post-transfection and pooled.

Cell lines were infected with virus-containing plain medium for 24 h. Medium was then replaced with puromycin containing medium to select for transduced cells, and incubated for 48 h. The MOI was determined at 72-h after infection by comparing the percent survival of infected cells to separate pools of cultured non-infected control cells. Optimal infection conditions were determined for each batch of virus prep in each cell line. Volumes of virus that yielded ∼10–30% infection efficiency were used for screening.

### Pooled screening and sequencing for sgRNA abundance

For all screens, cells with stable Cas9 expression were infected in three biological replicates per cell line with lentiviral sgRNA pools at a representation of 230–1000 cells per sgRNA at a MOI of 0.06 for Hs766T, 0.15 for HCC1428 and 0.30 for DLD1. MOI values varied by the infectivity of each cell line, but all obtained MOIs were below the target of 0.30. Cells were selected in the presence of puromycin, and a sample was collected 3 days post-selection as a reference representation of the pooled sgRNA library (except HCC1428). Cells were propagated for a total of 14 or 21 (HCC1428 only) days with an average representation of >1000 cells/sgRNA maintained at each passage. Cells were harvested for genomic DNA extraction (DNeasy Blood and Tissue kit, Qiagen Cat#69506). sgRNA inserts were first PCR amplified using primary PCR primers (Supplementary Table S20) on the vectors and purified by QIAquick PCR Purification Kit (Qiagen, Cat#28106). The PCR products from the genomic DNA were then further amplified using secondary PCR primers (Supplementary Table S20) harboring Illumina TruSeq adapters i5 and i7 barcodes. The 300 bp PCR product were purified by gel extraction (Qiagen, Cat#28706). The resulting fragments were sequenced on a MiSeq™ (Illumina) with standard primers for dual indexing. The sequencing recipe we used included 33 ‘dark cycles’ of base incorporation without imaging, followed by 21 light cycles with two indices.

### Screen analysis

Following sequencing, sgRNAs were counted with a custom pipeline based on Bowtie and Oculus (81,82). Reads per million mapped reads (RPMs) were computed for each sample, then normalized to the total abundance of guides targeting negative control genes (across libraries, such that the normalization factor for each cell line was the same) to account for variation between cell lines. sgRNA-level log2-fold-changes (LFCs), gene-level LFCs and gene-level coefficient of variation across sgRNAs (CV = standard deviation/mean) were computed with custom R code and gene-level beta values were computed with MAGeCK-VISPR’s maximum likelihood estimation model using negative control normalization (83). We plotted the distributions of counts in each gene category over time (Supplementary Figure S17). Next, we noted and plotted gene-level hit discrepancies between what we expected from Achilles versus what we observed. We defined an observed hit as reaching a mean dropout of more than 2-fold (LFC <−1) in at least one library. We included only genes both expected and observed to drop out in subsequent inter-library comparisons (‘hits’ in Figure 4 and Supplementary Figures S19 and S20). Finally, we assessed library performance using four different metrics: sgRNA LFCs, Gene LFCs, Gene-level beta values (from MAGeCK) and intra-gene CVs for all hits on a per-cell-line, per-library basis and plotted using custom R code (Supplementary Figure S19). *T*-tests were performed for each metric between each library and PINCER. We repeated this analysis with PINCER in a pseudo-4sgRNA/gene format, by analyzing only the top four sgRNAs selected by PINCER (picks i–iv) (Supplementary Figure S20). We also repeated this analysis for sgRNAs targeting negative control genes (olfactory receptors and solute carriers), omitting the negative control normalization step for both LFCs and MAGeCK, for PINCER in a six-guide format (Supplementary Figure S21) or four-guide format (Supplementary Figure S22).

## RESULTS

### Guide design strategy

**Figure 1.**
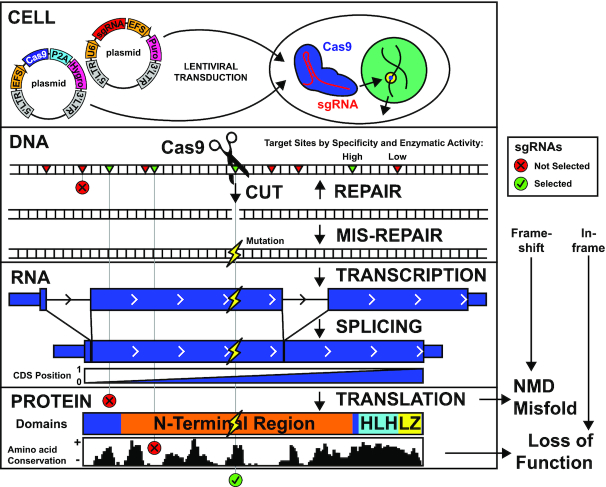
Maximizing Phenotypic Knockout with CRISPR/Cas9. Illustration of optimized CRISPR/Cas9 guide design for a typical target gene (MYC). An effective guide (green check) combines high Cas9 specificity and enzymatic activity with protein-level loss of function, considering both frameshift mutations outside the protein C-terminus and in-frame mutations in essential protein domains and conserved amino acids. NMD: nonsense-mediated decay, HLH: helix-loop-helix, LZ: leucine zipper.

While several genome-wide CRISPR libraries have been developed considering many guide features, no library yet has combined thermodynamic Cas9 enzymatic activity prediction (e.g. Doench *et al.* (13)) with gene feature targeting, particularly protein conservation (Supplementary Table S2). We set out to test which of the entire complement of features considered in guide design improve guide activity for pooled CRISPR screens. Figure 1 illustrates our strategy to combine guide-intrinsic and guide-target features for better guide prediction and ultimately improve pooled screens for target discovery. Briefly, guides and Cas9 DNA vectors are introduced into cells by lentiviral transduction. From many candidate positions on a gene, Cas9 is directed to those whose sequence context conveys high enzymatic activity and specificity. Cas9 nicks both DNA strands at the same position, effectively generating a blunt-ended double-stranded DNA break (DSB). The cell either repairs the DSB correctly (causing the process to repeat), or incorrectly to cause a mutation that propagates through transcription, splicing, and translation. Frame-shifting mutations are expected to disrupt protein function via NMD or protein misfolding unless mutations occur too close to the C-terminal of the mRNA transcript. However, in-frame mutations must be positioned within essential domains whose amino acid sequence is conserved in order to cause loss of protein function. We aim to select guides which maximize gene loss of function by considering each step of Cas9 editing.

### Comparison of cleavage efficacy and specificity scores in public sgRNA design algorithms

To assess the diversity of sgRNA cleavage efficacy and specificity scores, we downloaded all available MYC guide scores from all available tools which reported all sgRNAs for a gene: CHOPCHOPv2, CRISPOR (cleavage efficacy: Doench and Moreno-Mateos), CRISPR-DO, CRISPRko (GPP), GuideScan, GUIDES, sgRNA Scorer and compared them to the versions of cleavage efficacy (Doench *et al.*) and specificity (Hsu *et al.*) scores that we generated (12–13,42,45,84–87). We found only three unique cleavage efficacy scores amongst eight tools (Supplementary Figure S1 and Table S5) and three unique specificity scores with varied scaling among six tools (Supplementary Figure S2 and Table S6), with the most common ones being Doench *et al.* (13) (cleavage efficacy) and Hsu *et al.* (specificity) (12,13). Judging from these scores’ popularity and effectiveness in their original publications, we selected them for testing in our training analysis.

### A novel protein conservation score, AADelCons

While there exist several genome-wide nucleic acid conservation scores for the human and mouse genomes (e.g. PhastCons), to our awareness no such genome-wide score exists for protein conservation. Therefore, we evaluated conservation-based variant effect predictor tools commonly used to estimate the impact of somatic mutations (88). PROVEAN demonstrated a dramatic improvement over other algorithms in predicting the functional impact of BRCA1 RING domain mutations, approaching the predictive power of experimentally measured BRCA1:BARD1 binding affinity and E3 ligase activity (Supplementary Figure S5). PROVEAN also demonstrated a dramatic improvement over nucleotide conservation metrics in predicting sgRNA effectiveness (Supplementary Figure S6). By varying PROVEAN’s ortholog set and position-weight matrix, we found that the default parameters (BLAST NR and BLOSUM62) provided the best correlation with guide activity, especially when modeling single-amino acid deletions, which supports our strategy to target regions where even in-frame indels knock out gene function. As a further exploration of this idea, we found that deletions of varying lengths have similar predicted conservation scores (Supplementary Figure S23). Next, we generated a genome-wide conservation score by modeling every amino acid's deletion with PROVEAN as described in the methods. We verified that our conservation score is not highly correlated with cleavage efficacy, suggesting that the two metrics capture different aspects of what makes sgRNAs effective (Supplementary Figure S7).

### Amino acid conservation predicts sgRNA activity in three examples

We tested our new conservation score using data from three published experiments in which contextually essential genes were tiled by sgRNAs. In the first published experiment, the activities of sgRNAs targeting CD33, ANPEP and FUT4 were read out by abrogated antibody binding to their respective antigens CD33, CD13 and CD15 with flow cytometry (15). We found that sgRNAs targeting FUT4 demonstrated a stark relationship between target conservation and abrogated CD15 binding, but no such relationship existed for CD33 or ANPEP (Supplementary Figure S8). We learned that while the anti-CD33 and anti-CD13 antibodies target the direct protein products of the CD33 and ANPEP genes, CD15 is actually a fucosyl moiety that FUT4 transfers onto other proteins presented on the cell surface. We hypothesize that conservation did not predict guide activity for CD33 and ANPEP/CD13 because antibody recognition of a specific epitope is not necessarily related to target protein function. For instance, in-frame edits induced by a guide targeting a critical and conserved cytoplasmic domain could easily fail to disrupt antibody binding of an extracellular domain. In contrast, cell surface CD15 presentation is the phenotype of FUT4’s enzymatic activity, so sgRNA activity reflected the knockout of gene function and conservation had predictive value. This result clearly supports the motivation for targeting conserved residues to knock out gene function, particularly enzymatic function. Analysis of two additional published experiments also found a clear relationship between amino acid conservation and sgRNA activity (Supplementary Figures S9 and 10) (47,74). We note that while domain was a strong binary predictor, not all domains behaved the same—for instance, the POLR2A domain Herpes_BLLF1 (a herpes virus integration site) and Smarca4 domains Med15 and BRK are poorly conserved, and guides targeting them have little impact on cell survival (Supplementary Figures S9A and 10A). This underscores the heterogeneity of the label ‘domain,’ and suggests that amino acid conservation may serve as a better unbiased predictor in guide design.

### PINCER feature selection

**Figure 2.**
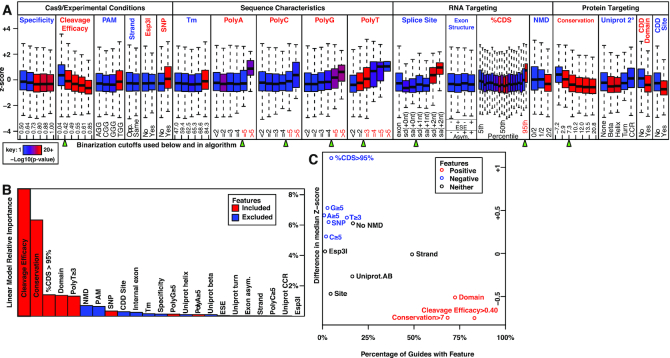
Guide design feature selection. Evaluation of which experimental, sequence, and targeting features best predict guide potency in a massive training dataset of contextually essential genes unbiasedly tiled by specific sgRNAs (*n* = 27 508 *z*-scored sgRNA-log2-fold-changes). (**A**) Boxplots of *z*-scores grouped by individual guide features, split logically for discrete features and into quintiles for continuous features, and colored by significance in *t*-tests to the left-most box of each feature. Specificity: Hsu *et al.* (12) score, Cleavage efficacy: Doench *et al.* (13) (‘Broad Rule Set 2’) score, PAM: protospacer adjacent motif, Strand: guide orientation relative to gene, Esp3I: restriction site, SNP: single nucleotide polymorphism in target at 10% VAF, *T*_m_: melting temperature, PolyA/PolyC/PolyG/PolyT: homopolymer length, Splice Site: cut site relative to exon edge in splice donors (sd) or splice acceptors (sa), exon structure: for targeted internal exons whether exon length is a multiple of 3 nt (asymmetric) and whether guide targets an exonic splicing enhancer (ESE) motif, %CDS: cut site relative to CDS, NMD: nonsense-mediated decay potential measured in number of frameshifts, Conservation: a deletion-based amino acid conservation score novel to this publication, Uniprot 2°: target's secondary structure if any (CCR: coiled-coil region), CDD Domain: conserved domain database domain targeted, CDD site: conserved domain database site targeted. Green arrows indicate binary cutoffs used in subsequent steps, and features used in the final algorithm are identified by red text. (**B**) Relative importance of each feature's contribution to a linear model predicting *z*-score. Bar colors indicate features included in the final design algorithm. (**C**) Difference in median *z*-score between guides possessing and lacking individual features versus the percentage of guides in the human genome possessing that feature. Uniprot.AB: guide targets either alpha helix or beta sheet. In addition to positive predictors from panel B (in red), this view highlights strong negative predictors not uncovered by the linear model (in blue).

We assembled a training dataset consisting of sgRNAs tiled on contextually-essential genes by combining seven datasets from five publications as described in the methods (*n* = 27 508 sgRNA-LFCs measurements) and tested each guide feature's ability to predict guide activity (Figure 2A, and in Supplementary Figure S11 with greater resolution for selected features). Results from Figure 2A are described from left to right. The specificity score cutoff we used (0.50, as suggested by both Haeussler *et al.* and Schoonenberg *et al.*) was sufficient to eliminate non-specific guides with high false activity due to off-target DNA damage, because guides with borderline specificity (0.50–0.64) did not demonstrate higher activity than others (41,89). Cleavage efficacy was a highly effective linear predictor of guide activity. The PAM site TGG was slightly less active than the other three (AGG, CGG, GGG), consistent with a previous report partially sharing the same training data (15). The relative orientation of gene and guide strands doesn’t impact guide activity, consistent with the idea that Cas9-induced double-stranded DNA breaks are blunt-ended and therefore strand-agnostic, and consistent with previous reports partially sharing the same training data (15,41). In these data, guides containing restriction sites were no less active than guides without restriction sites. Guides whose targets (23 nt) were affected by common single nucleotide polymorphisms (SNPs) and indels were less active in aggregate. sgRNA melting temperature (*T*_m_) had little impact on guide activity except that guides with very high *T*_m_ were slightly less active. Guides containing AAAAA, CCCCC, GGGGG, or TTT were less active than other guides. Guides targeting splice sites at the +0 (exon edge) and +1 (middle of splice site) positions were highly effective, but those targeting at +2 (splice site edge) were ineffective. Exon asymmetry and splicing enhancers had no impact on sgRNA activity. Guides targeting the 95th percentile of the protein CDS were less active than others, while guides targeting the 90th percentile were comparable to others in contrast with previous reports (see also Supplementary Figure S11B). Guides targeting positions in which 2/2 frameshifts induce NMD were more active than guides only capable of inducing NMD with 1/2 frameshifts or 0/2 frameshifts. Guides targeting conserved residues (score >7) were more active than those which did not target conserved residues. Guides targeting Uniprot beta sheets and alpha helices were more active than those which did not target predicted secondary structure. Guides targeting domains were more active than those which did not target domains. Guides targeting annotated regulatory sites in CDD were more active than those which did not target these sites. In particular, the best predictors of guide activity were cleavage efficacy and amino acid conservation. Although this data does not support the exclusion of guides containing restriction sites, we know from another study that these guides exhibit depressed counts prior to experiments being performed (Supplementary Figure S3). This effect is hypothetically caused by unintended restriction enzyme cleavage during cloning and results in increased noise and decreased sensitivity in screening.

On the basis of these observations, we selected binary cutoffs (Figure 2A, green arrows) and fit a linear model to predict z-score from continuous, categorical, and binary guide features. These are listed in order starting with Cleavage Efficacy, PAM and Esp3I (respectively) using the formula:

z ∼ Cleavage Efficacy + Specificity + Conservation + *T*_m_ + PAM + NMD + Esp3I + %CDS > 95% + ESE + Exon asymmetry + Internal Exon + Uniprot Beta + Uniprot CCR + Uniprot Helix + Uniprot Turn + PolyA ≥ 5 + PolyC ≥ 5 + PolyG ≥ 5 + PolyT ≥ 3 + Domain + SNP + CDD Site + Strand.

Respectively, the terms in the linear model represent: (z) *z*-score transformed observed guide-level log2-fold-changes; (Cleavage Efficacy) predicted guide cleavage efficacy on a zero to one scale as calculated by the Broad Rule Set 2 method; (Specificity) predicted guide specificity on a zero to one scale as calculated by the Hsu *et al.* (12) method; (Conservation) predicted protein conservation of the target residue on approximately as -7 to +21 scale, implemented using PROVEAN as defined in this manuscript; (Tm) predicted melting temperature of each guide as calculated by the HELP library; (PAM) the Protospacer Adjacent Motif of each guide; (NMD) whether each guide can induce NMD with zero, one, or two frameshifting frames; (Esp3I) whether guides and their flanking synthesized sequence contains an unintended restriction site; (%CDS > 95%) whether guides target the C-terminal end of the protein Coding Sequence; (ESE) whether guides target predicted Exonic Splicing Enhancers; (Exon asymmetry) whether guide target exons whose length is a multiple of 3 nt; (Internal Exon) whether guides target an exon that is not the first or last exon of the gene; (Uniprot Beta) whether guides target predicted beta sheets as defined by Uniprot; (Uniprot CCR) whether guides target predicted coiled-coil regions as defined by Uniprot; (Uniprot Helix) whether guides target predicted alpha helices as defined by Uniprot; (Uniprot Turn) whether guides target predicted turn regions as defined by Uniprot; (PolyA ≥ 5) whether guides and their flanking synthesized sequence contain AAAAA; (PolyC ≥ 5) whether guides and their flanking synthesized sequence contain CCCCC; (PolyG ≥ 5) whether guides and their flanking synthesized sequence contain GGGGG; (PolyT ≥ 3) whether guides and their flanking synthesized sequence contain TTT; (Domain) whether guides target predicted domains as defined by CDD; (SNP) whether guides and their PAMs overlap known point mutation or indel polymorphisms at a 10% VAF as defined by dbSNP; (CDD Site) whether guides target predicted critical annotated sites as defined by CDD; (Strand) whether guides are oriented in the same or opposite orientation of their target gene. For descriptions of how these scores were generated, see sections ‘Annotation of intrinsic guide features’ and ‘Annotation of guide target features’.

We then estimated variable importance using the relaimpo R package (90). We found that cleavage efficacy and conservation were not only the two most important predictors, but that they also complemented each other (Figure 2B). %CDS, Domain and PolyT ≥ 3 also contributed incrementally to the model. Repeating this analysis on a reduced set of linear model predictors uncovered no additional significant contributors (Supplementary Figure S25). Finally, to identify highly effective negative predictor features for a minority of sgRNAs, we plotted the difference in median *z*-score between guides with and without each feature versus the percentage of guides that each feature affects. This demonstrated the value of homopolymers and %CDS > 95% as negative selection criteria (Figure 2C).

We also considered that individual features may predict guide activity without complementing each other by competing for the same biological signal, so we assessed the interactions between biologically related features (Supplementary Figure S12). Conservation, domain and CDD Site actually do complement each other, though conservation is the best predictor and few guides target CDD Sites. However, conservation captures most of the signal that Uniprot features would otherwise contribute to the model. Surprisingly, %CDS > 95% is a dramatically better negative predictor of guide performance than the NMD-competency of guides. We hypothesize that non-NMD-inducing frame-shifting edits to the first 95% of the protein still alter the C-terminus enough to impair protein folding or function (Supplementary Figure S12D, leftmost box: %CDS > 95%: –, NMD: 0/2). No combination of splicing enhancer or exon symmetry features predicted dropout in this data. Supplementary Figure S24 illustrates the feature selection strategy.

### Algorithm and training performance

**Figure 3.**
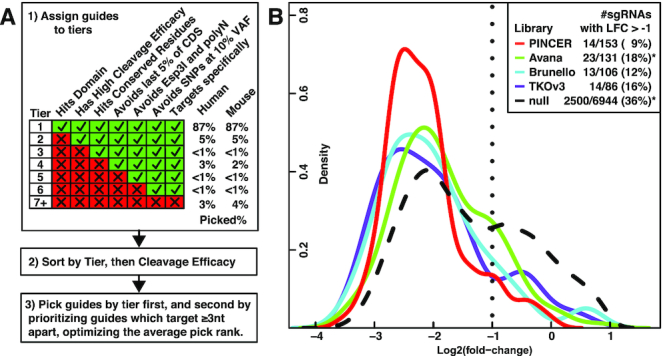
Guide Design Algorithm and Training. (**A**) Guide design algorithm. First, all guides in the genome are assigned to tiers by the features listed above the columns of the matrix. Features are ranked from left to right by expected predictive power and the percentage of guides they affect, such that highly effective predictors affecting a minority of guides are prioritized and specificity is the top priority. We were able to pick 87% of guides possessing all the listed features for both human and mouse. Next, guides are sorted first by their assigned tier, and then within tiers by cleavage efficacy (the best linear predictor of dropout). Finally, six guides per gene are picked to avoid overlaps and optimize rank. (**B**) Evaluation of retrospective performance of different libraries in the Munoz *et al.* (74), essential gene tiling training dataset (mean of three cell lines). sgRNAs which fail to achieve a 2-fold depletion (LFC < −1), are indicated by a dotted line, and asterisks indicate which libraries PINCER is significantly different from in terms of LFC distribution by *t*-test.

Using guide and target features selected in our training experiment, we devised an algorithm to pick any number of sgRNAs per gene (Figure 3A) and used it to generate six sgRNAs per gene to enable screens of either six sgRNAs/gene or four sgRNAs/gene, both of which have been proposed as optimal (37,91). All guides in the genome are assigned to tiers by whether they target domains, have high cleavage efficacy (>0.40), target conserved residues (score > 7), avoid the 3′ end of the CDS (%CDS < 95%), avoid Esp3I sites and homopolymers (CGTCTC, AAAAA, CCCCC, GGGGG, TTT), avoid common SNPs and indels (dbSNP, >10% VAF), and target specifically (Hsu *et al.* (12) > 0.50 and zero off targets with ≤1 mismatch). Tier 1 guides possess all these features and have the highest expected activity, and feature constraints are iteratively relaxed for all other tiers to enable targeting all genes. Specificity is the highest priority feature constraint (reflecting a strategic aim to minimize off-target activity), but if there aren’t enough specific guides for a gene, then that criterion is relaxed, and the process repeats as before (tiers 7.1–7.6) with 0.5 > specificity score >0.25, and finally removing the constraint entirely (tier 8—non-specific guides). We did not include features too rare to be feasible as inclusion criteria (CDD Site), too common or weak in effect to be feasible as exclusion criteria (PAM = TGG, high *T*_m_), too likely to cause unpredictable change of function variants (splice site), or those subsumed by other features (NMD, Uniprot secondary structure). Guides are then sorted by tier and cleavage efficacy (the best linear predictor of performance) and picked to maximize tier and rank with the added constraint of targeting ≥3 nt apart to limit overlapping guides. For instance, if a gene has three tier one guides, they are automatically included regardless of spacing, then three additional lower tier guides are selected to maximize their average rank around the 3 nt mutual spacing constraint. See Supplementary Table S21 for counts of sgRNAs in each tier. Supplementary Tables S22 and 23 contain PINCER in a six sgRNA/gene format, and Supplementary Tables S24 and 25 contain PINCER in a twelve sgRNA/gene format.

To retrospectively assess the training performance of our new library (PINCER), we compared the LFCs of guides shared between four libraries and the Munoz *et al.* tiling dataset, selected because it was the largest single dataset and was not used to fit any feature scores (e.g. cleavage efficacy is from Doench *et al.* (13)) (74). We averaged sgRNA observations across three cell lines, labeled sgRNAs shared by each library, and plotted their performance. Guides included in PINCER showed statistically stronger average knockout of essential genes by log2-fold-change compared to the null distribution and Avana (*P* < 0.001), and the lowest percentage of guides which fail to achieve 2-fold dropout (Figure 3B).

### PINCER validation in comparison to other libraries

We devised an experiment to directly compare PINCER’s performance to other genome-wide CRISPR libraries through a pooled CRISPR screen of select genes. First, we identified three cell lines from different cancer types with prior dropout data (Achilles 18Q3) and good Cas9 activity. These were the colorectal adenocarcinoma cell line DLD-1, pancreatic carcinoma cell line Hs766T and breast adenocarcinoma cell line HCC1428. Next, we identified genes to survey, which were expected to be either essential in all cell lines, essential in one or two of the three cell lines, or non-essential in all cell lines (see ‘Materials and Methods’ section and Supplementary Table S13 for the gene list). A total of 20 nt sgRNA sequences targeting pan-essential, cell line-essential and non-essential genes were pooled from Avana, Brunello, TKOv3 and PINCER, duplicates were removed, and a single pooled library was constructed. Using our database, we find that PINCER has slightly higher cleavage scores, similar specificity scores, a greater tolerance for 3’ guides and dramatically higher protein conservation scores than other libraries. However, the difference in conservation scores between PINCER and other libraries is reduced in the sub-library used in the validation experiment, hypothetically because essential genes tend to be highly conserved (Supplementary Figure S14). Overlaps between the libraries are minimal, so concordance between validation results cannot be attributed to shared guides between the libraries (Supplementary Figure S15).

**Figure 4.**
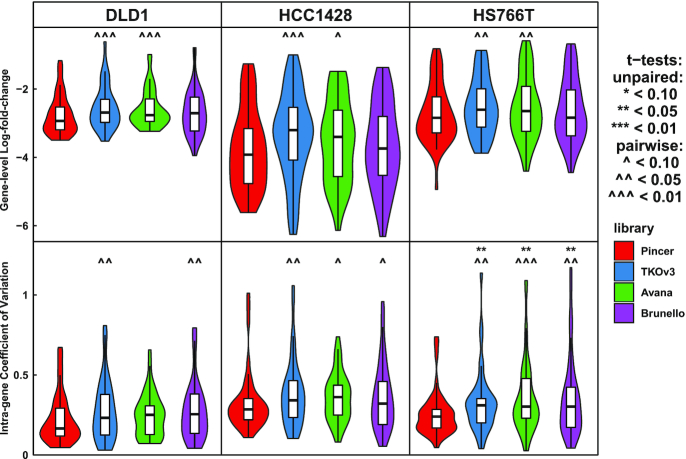
PINCER outperforms other libraries in effect size and robustness. CRISPR screens were conducted in the human cancer cell lines DLD1, HCC1428 and HS766T using pooled sgRNAs from four libraries (PINCER, TKOv3, Avana and Brunello) targeting pan-essential, selectively essential and negative control genes. Violin plots show gene-level log2-fold-changes, of reads per million mapped reads normalized to negative control reads (combining all libraries), between plasmid and endpoint for essential and cell-line-selective genes, and intra-gene coefficient of variation (across sgRNAs targeting the same genes), with paired and unpaired *t*-tests against the PINCER library.

We validated PINCER against other libraries by screening DLD-1, Hs766T and HCC1428 using the pooled sgRNA library in which all libraries’ guides shared the same vector and scaffold (see ‘Materials and Methods’ section; count and gene-level result data are available in Supplementary Tables S17 and 18). By T3, all genome-targeting sgRNAs depleted while non-targeting sgRNAs enriched, demonstrating the known DNA-damage effect of Cas9 editing (74,92). From T3 to T14 essential gene targeting sgRNAs depleted dramatically, while both targeting and non-targeting negative controls enriched (Supplementary Figure S17). Next, we identified a number of genes which were expected but not observed to drop out in any library and excluded them from comparative analysis between the libraries (Supplementary Figure S18). Finally, we directly compared dropout metrics between the four libraries for genes essential in each cell line (pan-essential genes + cell-line essential genes) (Figure 4; Supplementary Figures S19 and S20). PINCER displayed significantly stronger gene-level LFCs compared to TKOv3 and Avana in all cell lines tested, and statistically significantly reduced intra-gene variation (robustness) compared to all three other libraries in all but one out of nine comparisons.

## DISCUSSION

In this work, we surveyed a wide array of proposed guide design features for CRISPR/Cas9 screening and empirically determine which features best predicted guide activity and how best to combine them. In particular, we anticipated that the combination of predicted Cas9 cleavage efficacy and target features important to protein function would be especially effective.

Targeting sgRNAs to essential domains emerged as a successful strategy to screen for cancer drug targets in 2015, and it seems clear in retrospect that edits to non-essential regions of essential genes could easily fail to produce phenotypic changes in gene function (47). Cas9 induces both frameshift edits and in-frame edits. While frameshifts are highly likely to knock out gene function by either cellular surveillance mechanisms or protein-level dysfunction, the impact of in-frame indels and point mutations depends highly on their positions in the protein. Moreover, as others have noted, an expected indel frameshift rate of two-thirds implies a biallelic frameshift rate of only four/ninths (41). Obtaining frameshifts in all alleles is even less likely for copy-gained genes, which are common in cancer and driven lower still by point mutation edits. Taken together, Cas9-induced in-frame edits are probably very common.

We reasoned that targeting conserved protein regions could not only solve ineffective in-frame edits, but also improve on domain targeting in four ways. First, while domains are sparsely annotated for poorly characterized gene families, conservation provides unbiased annotations for all genes in the genome. This serves to approximate the genome-wide structure-function knowledge others have noted domain targeting lacks (93). Second, conservation at single-residue resolution is more precise than domain annotations, enabling targeting of critical regions even within domains whose conservation varies widely (e.g. FUT4’s N-terminal fucosyltransferase domain, Supplementary Figure S8C). Third, domain annotations have heterogeneous meanings, and often don’t represent functional gene subunits (e.g. the herpesvirus integration site in POLR2A, Supplementary Figure S9A). Finally, by modeling in-frame deletions, protein regions whose lengths are variable across evolution can be distinguished from protein regions whose lengths are highly conserved. Observing conservation in the length of a protein region across evolution implies that its length is under purifying selection, and that the region's structure may require that specific fixed length in order to function. Conserved-length regions make for excellent CRISPR targets because even in-frame indels are likely to disrupt their function.

PROVEAN’s elegant approach to predict the functional impact of deletions is to compare the respective alignments of a gene's wild-type and mutant proteins to the gene's orthologs. In-frame deletions do not impact alignment to gene regions whose lengths are variable across evolutionary history (scoring low), but highly disrupt alignment to regions whose lengths are invariable (scoring high), resulting in bimodality of our genome-wide conservation score (Supplementary Figure S11A). Because the purpose of targeting Cas9 to functional regions is to ensure that in-frame edits disrupt gene function, capturing this invariable-length component of conservation is a significant improvement. We are the second to propose this approach after Schoonenberg *et al.*, and the first to include it in a genome-wide library design (41). Others have proposed conservation targeting based on amino acid substitutions or nucleotide conservation, but we demonstrate here that PROVEAN’s model likely improves variant effect prediction (Supplementary Figure S5) and guide activity prediction (Supplementary Figure S6) over those alternatives. To the best of our knowledge, our work also produced the first genome-wide protein conservation track (AADelCons), which likely has utility beyond CRISPR guide design and is available on Github.

As a first validation of our design strategy, we observed that cleavage efficacy and conservation were individually highly predictive of guide activity in aggregate, although both have wide variation (Figure 2A). They are not highly correlated (Supplementary Figure S7) and complement each other effectively to predict overall guide activity (Figure 2B). Conservation subsumed the individual predictive value of protein secondary structure, and to a lesser extent CDD site and CDD Domain which still added value (Supplementary Figure S12A–C). The observations that deletion-based protein conservation predicts guide activity and is complemented by domain annotations were also made by Schoonenberg *et al.* in training data partially overlapping this experiment. Further, differences were small between simulated deletions of varying length (Supplementary Figure S23), likely owing to autocorrelation by considering overlapping amino acids but also because all short deletions capture the same ‘conserved length’ signal.

Unexpectedly, we observed that guides containing thymine trinucleotides (TTT) exhibit lower activity (Figure 2A), even in combination with cleavage efficacy (Figure 2B). This is significant because most algorithms only omit guides containing length four (TTTT), believing that to be the minimum required for Pol III termination as supported by experimental data (29). Impaired sgRNA activity by TTT has also been observed at least once before, so it is possible that our observation is consistent with TTT-mediated Pol III transcription termination (19). Critically, the commonly used ‘F+E’ sgRNA scaffold (Chen *et al.*) and recently improved LRG2.1 scaffold (Tarumoto *et al.*) contain two TTTs and three TTTs respectively, implying it may be possible to improve sgRNA expression for any application by further scaffold modifications (53,54).

PINCER guides demonstrated significantly stronger intra-gene concordance than those of Avana, TKOv3 and Brunello in most comparisons. We anticipate that this improvement will benefit gene-level significance testing in screens, in which a minority of discordant guides can really impair sensitivity. However, while PINCER showed significantly stronger gene-level activity than Avana and TKOv3, the improvement in gene-level activity over Brunello was not significant. There are two possible technical limitations of our experiment which could explain this. First, because we were testing multiple libraries in multiple cell lines, the number of genes surveyed was necessarily low (44–46 essential genes per cell line), and it's possible that comparing more genes between PINCER and Brunello could better delineate their differences. Second, while PINCER demonstrates a dramatic difference from other libraries in guides targeting conserved sequence genome-wide (96 versus 53, 54, 60%; see Supplementary Figure S14A, upper-right panel), that separation is reduced in the genes surveyed in our experiment (100 versus 72, 74, 79%; see Supplementary Figure S14A, lower-right panel). Because essential genes tend to be entirely conserved, it is possible that by testing essential genes this experiment underestimated the benefits of conservation targeting. We also expect that even modest improvements in guide activity will benefit screening sensitivity, as the difference between an 80 and 90% effective guide rate corresponds probabilistically to 81.9 versus 94.8% of genes with at least three/four effective guides, 65.5 versus 88.6% of genes with at least five/six effective guides and 90.1 versus 98.4% of genes with at least four/six effective guides, assuming effective guides are distributed randomly with respect to genes.

While this work was ongoing, improvements were made to established scoring methods and additional guide design features were proposed. Novel predictors of cleavage efficacy and specificity from guide sequence were proposed (Supplementary Table S4). The most promising of these incorporate either principled energy simulations or machine learning using large training datasets. Incorporating updated scores into our model could likely improve guide activity. Promising novel guide design features include predicted sgRNA scaffold misfolding, misrecognition of Cas9 to excessive PAM sites near the guide target, misrepair prediction by cut site sequence context (microhomology), target chromatin accessibility, isoform expression and somatic variants in the target (Supplementary Table S3) (17,26,31–32,42,94). However, we expect that most of these depend on cellular context. Chromatin accessibility and isoform expression are famously variable between cell lines and lineages. Every cancer cell line has a unique set of somatic mutations. The fact that specific DNA repair pathway deficiencies cause consistent mutation patterns across patients (so-called ‘mutational signatures’) implies that Cas9-induced mutation patterns probably also depend on DNA repair pathway status (95). Moreover, mutation patterns are likely to vary highly across cell lines as DNA repair genes are among the most frequently disrupted in cancer (e.g. TP53), and may be especially difficult to predict in the absence of DNA repair deficiencies as relatively normal cells exhibit erratic CRISPR editing (35). Still, the evidence is compelling that these features hold predictive value within their respective contexts. While it is difficult to imagine incorporating these into genome-wide library design generally, the right application of these features may be cellular-context-aware screen analysis as suggested in CRISPRO (41). It may be possible to reach even higher levels of sophistication, for instance by using PROVEAN to predict the functional impact of the distribution of predicted mutations at a cut site (linking misrepair prediction to conservation analysis). Finally, it has been observed that cells can compensate for gene loss by upregulating paralogs (e.g. ‘transcriptional adaptation’ mechanistically linked to NMD), and some groups have proposed simultaneous targeting of multiple paralogs (e.g. CRISPys) (96,97). However, as this approach requires either prior knowledge of paralog sets and/or expanding library size, it remains a limiting factor for single-guide libraries like PINCER.

Here we present the PINCER genome-wide CRISPR library, which for the first time combines Cas9 cleavage efficacy optimization with deletion-based protein conservation targeting. PINCER was trained using the most comprehensive feature database and the largest training dataset yet assembled. PINCER guides achieve superior performance compared to other libraries. The human and mouse PINCER libraries presented here will improve drug target discovery screens both *in vitro* and *in vivo*, empower cell and animal engineering to study individual gene function, and could even drive direct Cas9 therapies in the future.

## DATA AVAILABILITY

The human and mouse genome-wide PINCER libraries, guide databases, conservation scores and all code are available on Github: (https://github.com/veeneman/PINCER).

## Supplementary Material

gkaa645_Supplemental_Files
