# Supplementary material for: PINCER: improved CRISPR/Cas9 screening by efficient cleavage at conserved residues
Source: Nucleic Acids Res. 2020 Aug 21;48(17):9462–77. doi: 10.1093/nar/gkaa645 (PMC7515706; doi:10.1093/nar/gkaa645)

SUPPLEMENTARY TABLE AND FIGURE LEGENDS

Supplementary Table 1. Review of published CRISPR guide design literature reviews.

Supplementary Table 2. Review of published CRISPR libraries.

Supplementary Table 3. Review of published CRISPR guide design tools.

Supplementary Table 4. Review of published CRISPR guide scoring algorithms.

Supplementary Table 5. Cleavage efficacy scores from guide design tools for the human gene MYC, used to generate Supplementary Figure 1.

Supplementary Table 6. Specificity scores from guide design tools for the human gene MYC, used to generate Supplementary Figure 2.

Supplementary Table 7. Variant effect predictions and HDR competency of RING domain mutations of the human gene BRCA1, used to generate Supplementary Figure 5.

Supplementary Table 8. Manually curated Homologene ortholog sets for the human genes PLK1 and MED12, and the mouse gene Smarca4.

Supplementary Table 9. Nucleotide and protein conservation scores for the human genes PLK1 and MED12, and the mouse gene Smarca4, used to generate Supplementary Figure 6.

Supplementary Table 10. Summary statistics of the seven datasets used to generate the aggregate model training dataset used in this publication.

Supplementary Table 11. Column descriptions of sgRNA features used in model training.

Supplementary Table 12. Model training data. See Supplementary Table 11 for column descriptions.

Supplementary Table 13. Final list of genes used in the validation experiment.

Supplementary Table 14. Scores used to identify essential genes for the validation experiment.

Supplementary Table 15. Scores used to identify CL-selective genes for the validation experiment.

Supplementary Table 16. Scores used to identify non-essential genes for the validation experiment.

Supplementary Table 17. sgRNA counts in the validation experiment, with annotated features.

Supplementary Table 18. Gene-level changes in the validation experiment.

Supplementary Table 19. Illustrated sgRNA oligomer cloning diagram.

Supplementary Table 20. PCR primers.

Supplementary Table 21. Counts of sgRNAs in PINCER in each guide tier.

Supplementary Table 22. PINCER Human Library in 6 sgRNA/gene format.

Supplementary Table 23. PINCER Mouse Library in 6 sgRNA/gene format.

Supplementary Table 24. PINCER Human Library in 12 sgRNA/gene format.

Supplementary Table 25. PINCER Mouse Library in 12 sgRNA/gene format.

Supplementary Table 26. Nucleotide and protein conservation scores for the human genes PLK1 and MED12, and the mouse gene Smarca4, used to generate Supplementary Figure 23.

Supplementary Figure 1. Guide design tools use few unique cleavage efficacy scores. (A) Correlation heatmap and (B) scatterplots of cleavage efficacy scores from all available MYC sgRNAs for eight public sgRNA algorithms and PINCER. crispor.mm: the Moreno-mateos score in CRISPOR, crispor.doench: the Doench '16 score in CRISPOR.

Supplementary Figure 2. Guide design tools use few unique specificity scores. (A) Correlation heatmap and (B) scatterplots of specificity scores from all available MYC sgRNAs for six public sgRNA algorithms and PINCER.

Supplementary Figure 3. Guides containing restriction sites are depressed in Achilles. Scatterplot of log10(total sgRNA counts +1) in the Broad Achilles project (19Q1, sum of cell lines). sgRNAs are ordered alphabetically by sequence, and guides starting with the Esp3I/BsmbI restriction site sequence (CGTCTC) are colored in red.

Supplementary Figure 4. Primer3 and calcTm produce similar sgRNA melting temperatures. Scatterplot comparing melting temperature predictions for the model training data, using Primer3 (X-axis) and calcTm from the HELP Bioconductor library (Y-axis).

Supplementary Figure 5. PROVEAN is a superior variant effect prediction tool. Scatterplots comparing homology-dependent repair efficiency measurements for BRCA1 (HDR, Y-axis on all panels) to (A) an experimentally trained prediction, (B-E) variant effect predictions from a series of algorithms, and variant effect predictions from PROVEAN using either (F) a web app, (G) locally using BLAST NR 2017, or (H) locally using BLAST NR 2011. The HDR, HDR_predictions, SIFT, Polyphen, CADD, and GERP data come from a previously published study (1). (I) Significance tests (R's cor.test) of the correlation between each prediction and HDR, using Pearson's correlation coefficient, Spearman's rho, and Kendall's tau.

Supplementary Figure 6. Deletion-based protein conservation better correlates with dropout than either substitution-based protein conservation or any form of nucleotide conservation. Heatmap and unsupervised clustering of Spearman correlation values between different conservation scores (X-axis labels) and published sgRNA depletion for essential genes (PLK1, Smarca4) or enrichment for a synthetic resistance gene (MED12). Parameters tested for PROVEAN included the ortholog set: BLAST NR 2011 (NR), BLAST NR 2011 with primate and synthetic orthologs pruned (NRp), and a manually

curated ortholog set from NCBI Homologene (HG), the position-weight matrices (PWM) BLOSUM62 (BL) and the identity matrix (ID), and for variants the effect of single amino-acid deletions (Del) and substitutions (Sub). A green arrow indicates the parameter set used to generate our genome-wide score.

Supplementary Figure 7. Conservation and Cleavage efficacy (the Broad Rule Set 2 score) are distinct. 2d heatmaps showing the relationship between cleavage efficacy (the Broad Rule Set 2 score) and our novel conservation score for either (A) the whole human genome, or (B) guides used in the training dataset.

Supplementary Figure 8. Tiling Anecdote #1/3 – Conservation predicts flow-based dropout of guides targeting the enzyme FUT4 and not the antigens CD33 or CD13. Log2-fold-changes (LFCs) from a published experiment in which depletion of tiled sgRNAs was measured by flow cytometry using antibodies for (A,B,D,E) target genes or (C,F) the target gene's metabolite (2). (A-C) Integrative Genomics Viewer (IGV) screenshots of the target genes, their subcellular localization, their domains, our protein conservation score, and per-sgRNA LFCs for three cell lines (where available). Vertical red bars indicate cropped introns. (D-F) scatterplots comparing conservation to LFC.

Supplementary Figure 9: Tiling Anecdote #2/3 – Conservation and cleavage efficacy predict guide activity against the pan-essential gene POLR2A. Log2-fold-changes (LFCs) from a published experiment in which depletion of tiled sgRNAs was measured by cellular proliferation in a pooled screen. (A) IGV genome browser screenshot illustrating the gene POLR2A (RNA Pol II Subunit A), its domains, conservation score, and per-sgRNA LFCs for three cell lines. We derived per-gene, per-CL z-scores from the LFCs. Scatterplots comparing average z-score for the three cell lines to (B) conservation and (C) cleavage score (Broad Rule Set 2), and (D) as boxplots split around binary guide features.

Supplementary Figure 10. Tiling Anecdote #3/3 – Conservation, cleavage efficacy, and domains predict guide activity against the selectively-essential gene Smarca4. Log2-fold-changes (LFCs) from a published experiment in which depletion of tiled sgRNAs was measured by cellular proliferation in a pooled screen (3). (A) Integrated Genomics Viewer screenshot illustrating the mouse gene Smarca4, its domains, its conservation, and per-sgRNA LFCs and z-scores. Red bars indicate cropped introns, and arrows indicate a wrap-around for graphing purposes. Scatterplots of LFC (Y-axis) *vs.* (B) conservation and (C) cleavage score, and (D) boxplot of LFC vs. domain.

Supplementary Figure 11. Individual plots showing conservation, domains, %CDS > 95%, and homopolymers predict dropout in an aggregate training dataset. Depletion z-scores for specific sgRNAs tiling essential genes, aggregated from five publications (see methods section "Training dataset"). (A) Scatter plot and boxplot comparing z-score to conservation and domain. (B) Scatter plots comparing the position of each guide's edit site in the transcript (%CDS) to guide dropout, with moving average lines binned as labeled, including either all genes in the training dataset (above), or genes longer than two kilobases (below). (C) Boxplots showing the relationships between all guides containing homopolymers of minimum lengths to dropout (in contrast to Figure 2, these are all greater-than-or-equal-to groups).

Supplementary Figure 12. In predicting dropout, Conservation supersedes CDD Site, Uniprot annotations, and not Domains, %CDS supercedes NMD, and Exon asymmetry is ineffective. Interactions between conceptually related guide features *vs.* sgRNA log2-fold-change z-score in the training dataset. (A) Boxplots of conservation and domain-targeting status, *vs.* z-score. (B) Boxplots of conservation and CDD site *vs.* z-score. (C) Boxplots of conservation and Uniprot secondary protein structure annotations *vs.* z-score. (D) Boxplots illustrating %CDS and nonsense-mediated decay competency of each guide (#NMD-inducing-frameshifts) *vs.* z-score. (E) Boxplots illustrating exon asymmetry and exonic splicing enhancers *vs.* z-score.

Supplementary Figure 13. RNAi and CRISPR identified similar essential genes. Scatterplot comparing mean essentiality score from a CRISPR-based publication of essential genes (Hart '17, X-axis) to significance (Y-axis). Point colors indicate whether genes were identified as essential in the CRISPR publication (red), an RNAi-based publication of essential genes (blue), or both (black). A horizontal dashed line indicates the significance cutoff used for gene selection.

Supplementary Figure 14. Guide library feature distributions. Distributions of feature scores in our sgRNA database for four genome-wide libraries, either in their entirety (A above, B left), or the subset of their guides included in our validation experiment (A below, B right).

Supplementary Figure 15. Guide libraries do not significantly overlap. Venn diagrams showing the number of guides in common between each of (A) four genome-wide CRISPR libraries, and (B) the guides from those libraries included in our validation experiment.

Supplementary Figure 16. Cloning vector diagrams. Feature maps of our (A) sgRNA and (B) Cas9 cloning vectors.

Supplementary Figure 17. Summary statistics of validation experiment sample composition. Stacked barplot showing the fraction of sgRNA counts targeting genes in eight categories (colors labeled on the right) in each replicate of our validation experiment (see methods). Gene groups include non-targeting controls (nt.ctrl), negative control olfactory receptors (OR) and solute carriers (SLC), cell-line selective kinases and transcription factors (TF), and essential genes with broad and narrow domain architectures.

Supplementary Figure 18. Identification of genes both expected and observed to drop out in validation experiment. Heatmap of mean LFCs of all guides from all libraries (cell colors) targeting each gene in our validation experiment (X-axis) in each cell line (Y-axis). Markers indicate whether a gene was expected to drop out, and whether it was observed to drop out by at least two-fold in at least one library. For subsequent analysis of the validation dataset, we used only genes both expected and observed in one library to drop out.

Supplementary Figure 19. Head-to-head validation of the PINCER library (6 sgRNAs/Gene) *vs.* three public libraries, for essential genes. Violin plots showing performance of each of four libraries in our pooled head-to-head validation experiment. The three main columns correspond to the three cell lines tested, and the four main rows correspond to each of four different metrics of library performance. Within

each comparison are violin plots comparing guides targeting pan-essential and cell-line-essential genes in PINCER to the other three libraries, with unpaired t-tests labeled in asterisks, and paired t-tests labeled in carets (except for sgRNA dropout, for which there aren't pairs). LFCs were computed as the log2-fold change between reads per million mapped reads normalized to negative control reads (combining all libraries), at endpoint *vs.* plasmid, and MAGeCK beta values were also normalized to negative control genes.

Supplementary Figure 20. Head-to-head validation of the PINCER library (4 sgRNAs/Gene) *vs.* three public libraries, for essential genes. Violin plots showing performance of each of four libraries in our pooled head-to-head validation experiment, with the additional step of considering only four sgRNAs from the PINCER library (picks #1-4). The three main columns correspond to the three cell lines tested, and the four main rows correspond to each of four different metrics of library performance. Within each comparison are violin plots comparing guides targeting pan-essential and cell-line-essential genes in PINCER to the other three libraries, with unpaired t-tests labeled in asterisks, and paired t-tests labeled in carets (except for sgRNA dropout, for which there aren't pairs). LFCs were computed as the log2-fold change between reads per million mapped negative control gene reads (combining all libraries) at endpoint *vs.* plasmid, and MAGeCK beta values were also normalized to negative control genes.

Supplementary Figure 21. Head-to-head validation of the PINCER library (6 sgRNAs/Gene) *vs.* three public libraries, for negative control genes. Violin plots showing performance of each of four libraries in our pooled head-to-head validation experiment. The three main columns correspond to the three cell lines tested, and the four main rows correspond to each of four different metrics of library performance. Within each comparison are violin plots comparing guides targeting non-essential genes in PINCER to the other three libraries, with unpaired t-tests labeled in asterisks, and paired t-tests labeled in carets (except for sgRNA dropout, for which there aren't pairs). LFCs were computed as the log2-fold change between reads per million mapped reads at endpoint *vs.* plasmid, and MAGeCK beta values were median normalized.

Supplementary Figure 22. Head-to-head validation of the PINCER library (4 sgRNAs/Gene) *vs.* three public libraries, for negative control genes. Violin plots showing performance of each of four libraries in our pooled head-to-head validation experiment, with the additional step of considering only four sgRNAs from the PINCER library (picks #1-4). The three main columns correspond to the three cell lines tested, and the four main rows correspond to each of four different metrics of library performance. Within each comparison are violin plots comparing guides targeting non-essential genes in PINCER to the other three libraries, with unpaired t-tests labeled in asterisks, and paired t-tests labeled in carets (except for sgRNA dropout, for which there aren't pairs). LFCs were computed as the log2-fold change between reads per million mapped reads at endpoint *vs.* plasmid, and MAGeCK beta values were median normalized.

Supplementary Figure 23. Conservation scores for deletions of varying lengths. For each of three example genes (panels A, B, C: PLK1, MED12, Smarca4), PROVEAN was used to predict the effect of the deletion of every single amino acid (1AA del), every adjacent pair of amino acids (2AA del), and

every adjacent trio of amino acids (3AA del). Plotted are the conservation scores of individual amino acids, for every deletion affecting them in every sliding window.

Supplementary Figure 24. Schematic illustrating feature selection and model building, relating to Figures 2 and 3. (A) sgRNA training data was annotated by sgRNA features and filtered to specific sgRNAs, such that observed sgRNA effect can be attributed solely to on-target factors. sgRNAs targeted to splice sites were analyzed independently, and the remaining data was analyzed within the context of exonic sgRNAs. (B) First, the individual predictive value of every feature was assessed, and binary cutoffs were identified (Figure 2A) and applied as indicated in Figures 2B and 2C. Second, the additive value of features was assessed (Figure 2B) to identify five key additive predictors of sgRNA activity. Third, the predictive values of all binary features were assessed as exclusion criteria (Figure 2C). Fourth, supervised analysis of biologically related features was performed to clarify which had pairwise additive value and which were superseded (Supplementary Figure 12). Finally, the features which had individual predictive value and either additive or minority predictive value and were not superseded by other features were identified. To this list, we manually added Esp3I restriction sites and splice sites as exclusion criteria, based on prior experience (Supplementary Figure 3) and a desire to avoid change-of-function variants. (C) First, selected features were ranked by their predictive value and the number of sgRNAs they affected and used to assign sgRNAs to tiers as indicated in Figure 3. In this way, relatively less-effective predictors are sacrificed first, followed by more-effective predictors, and finally followed by specificity. Next, guides are ranked by tier first, cleavage efficacy second (the best linear predictor), and finally picked with a 3nt spacing criterion to optimize average rank.

Supplementary Figure 25. Relative importance of linear predictors, relating to Figure 2B. A linear model was used to predict z-score in the training data experiment as in Figure 2B, combining the five features Cleavage Efficacy, Conservation, %CDS>95%, Domain, and PolyT>=3 (top-left panel), and adding a varying sixth feature (remaining panels). Each panel shows the results of a separate linear model. Y-axis values are relative variable importance as calculated using the relaimpo Bioconductor package (LMG method).

REFERENCES

1. Starita, L.M., Young, D.L., Islam, M., Kitzman, J.O., Gullingsrud, J., Hause, R.J., Fowler, D.M., Parvin, J.D., Shendure, J. and Fields, S. (2015) Massively Parallel Functional Analysis of BRCA1 RING Domain Variants. Genetics, 200, 413-422.
2. Doench, J.G., Hartenian, E., Graham, D.B., Tothova, Z., Hegde, M., Smith, I., Sullender, M., Ebert, B.L., Xavier, R.J. and Root, D.E. (2014) Rational design of highly active sgRNAs for CRISPR-Cas9-mediated gene inactivation. Nat Biotechnol, 32, 1262-1267.
3. Shi, J., Wang, E., Milazzo, J.P., Wang, Z., Kinney, J.B. and Vakoc, C.R. (2015) Discovery of cancer drug targets by CRISPR-Cas9 screening of protein domains. Nat Biotechnol, 33, 661-667.

Supplementary Figure 1

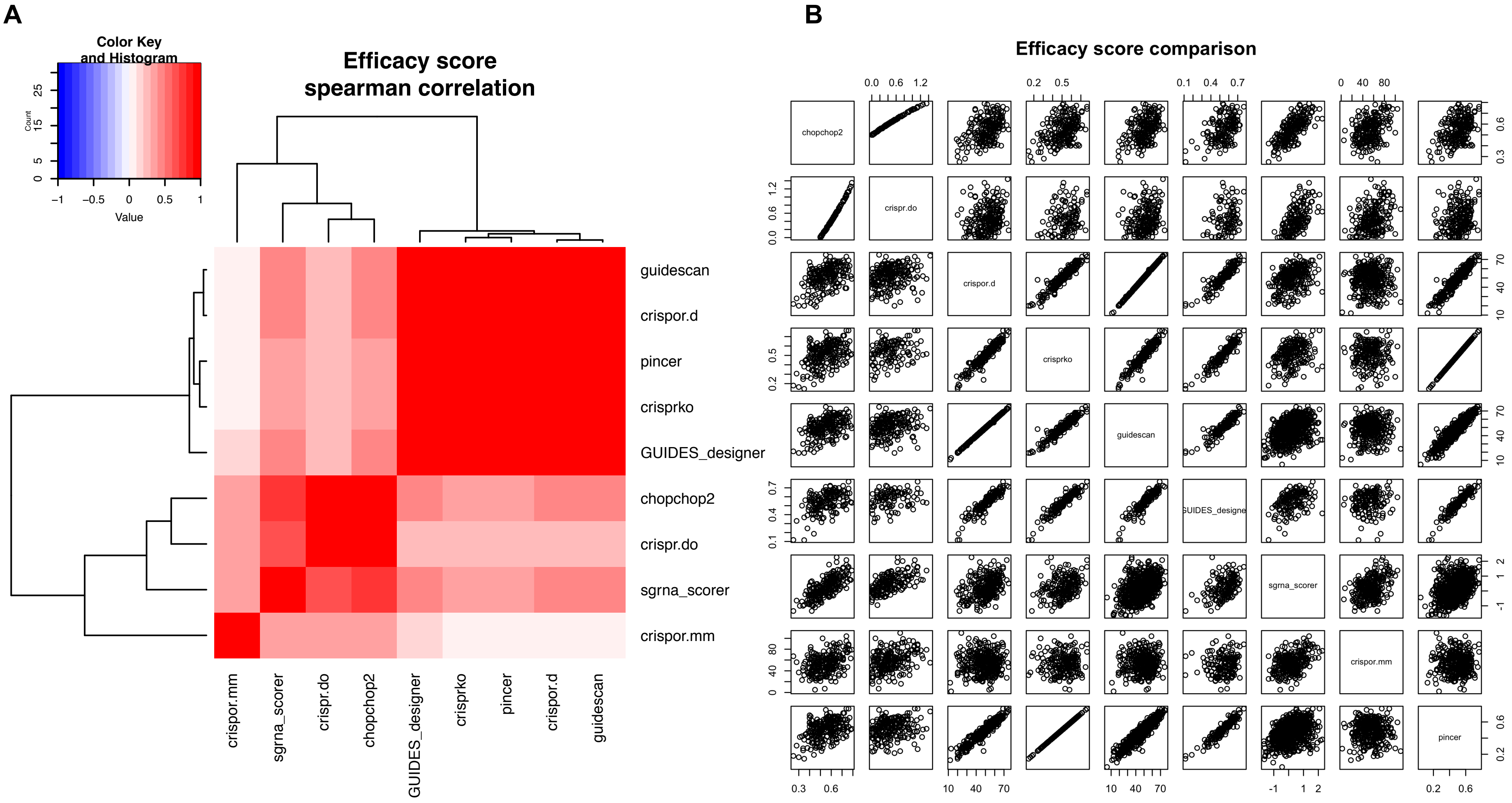

Supplementary Figure 2

A

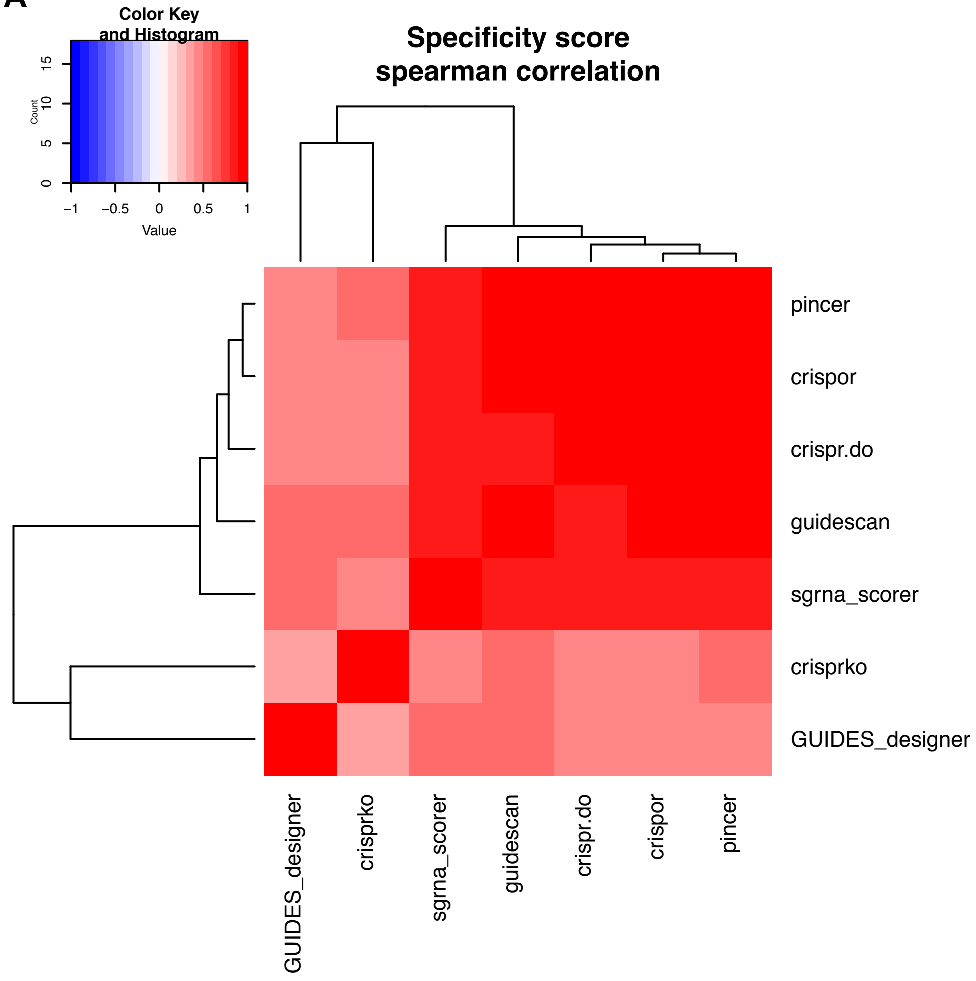

B

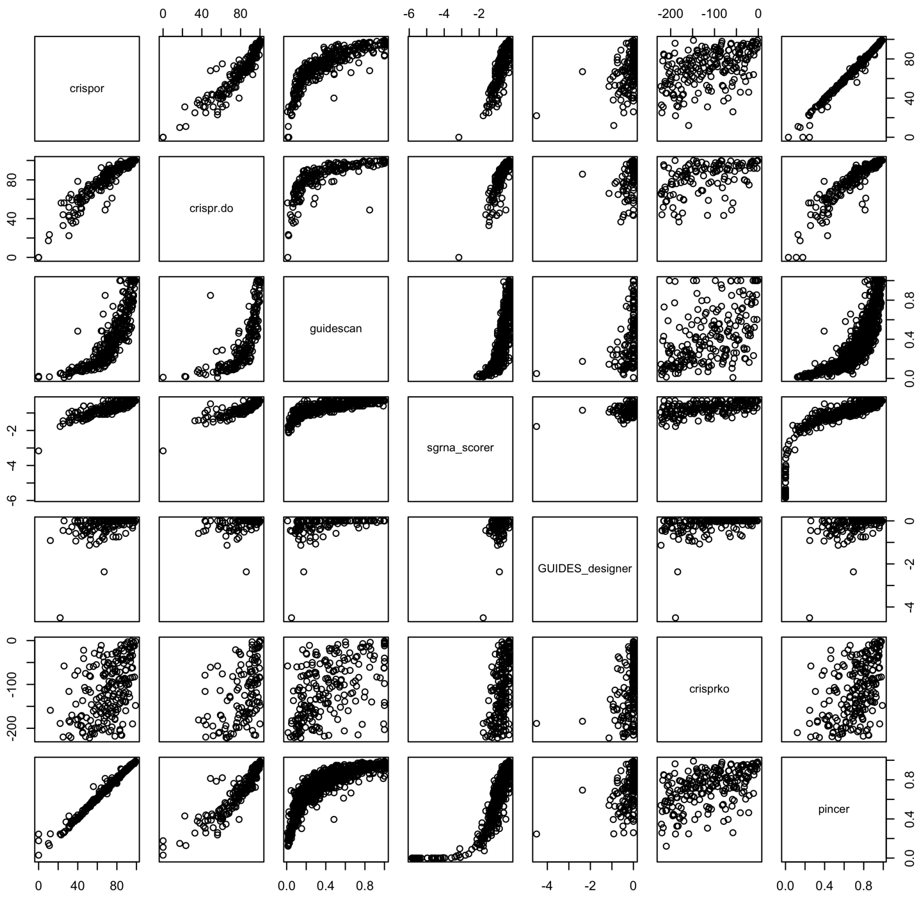

Supplementary Figure 3

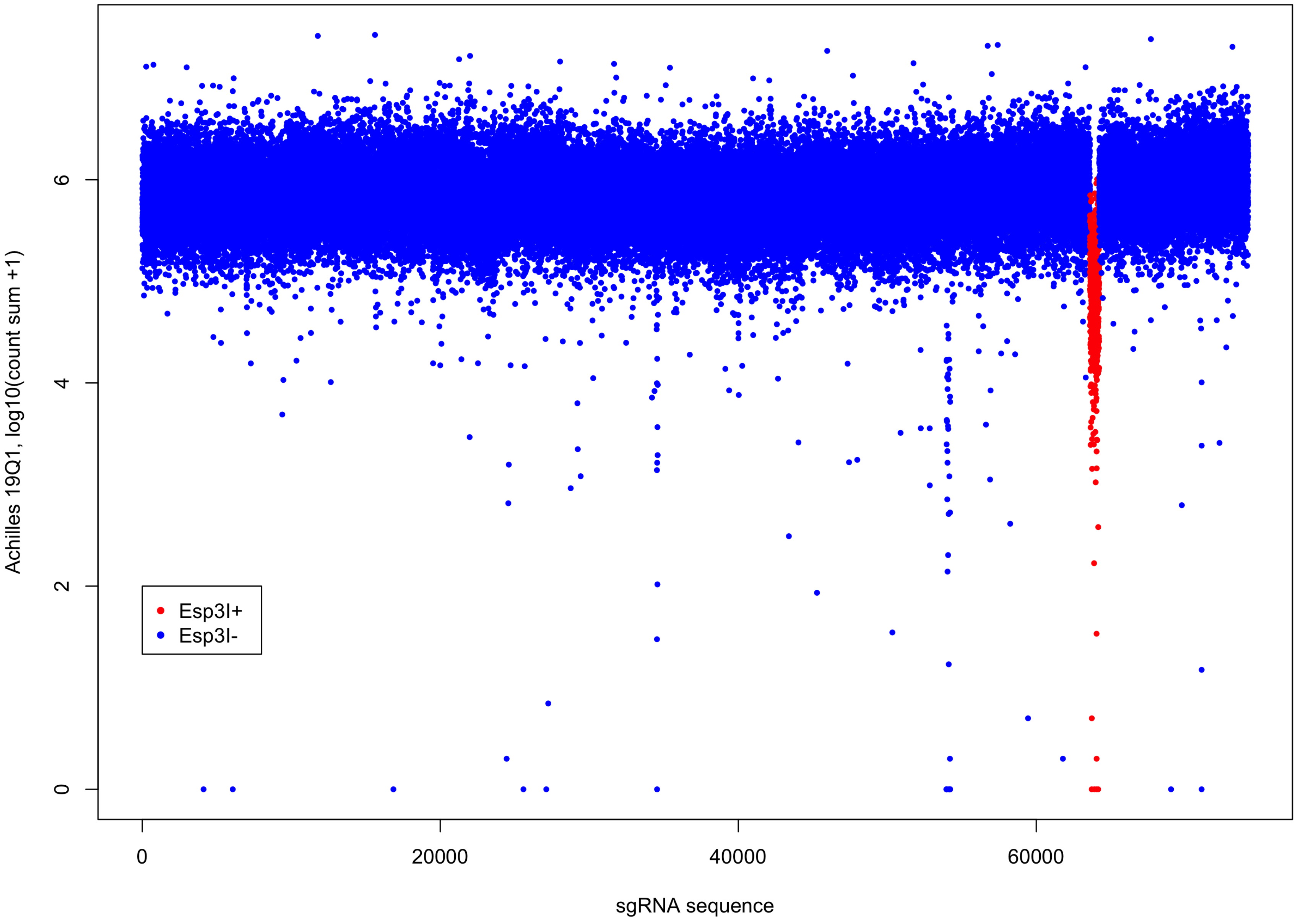

Supplementary Figure 4

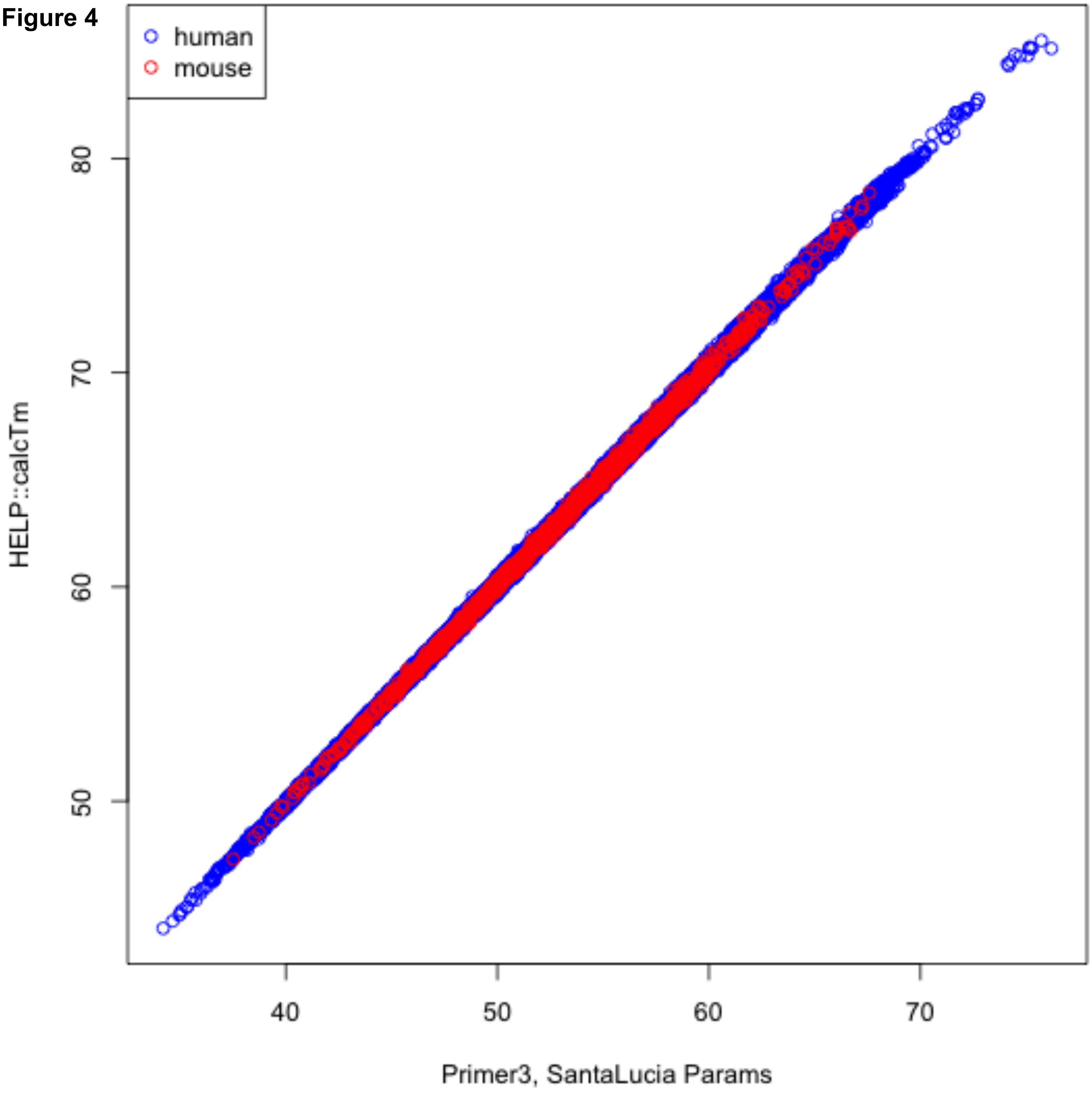

Supplementary Figure 5

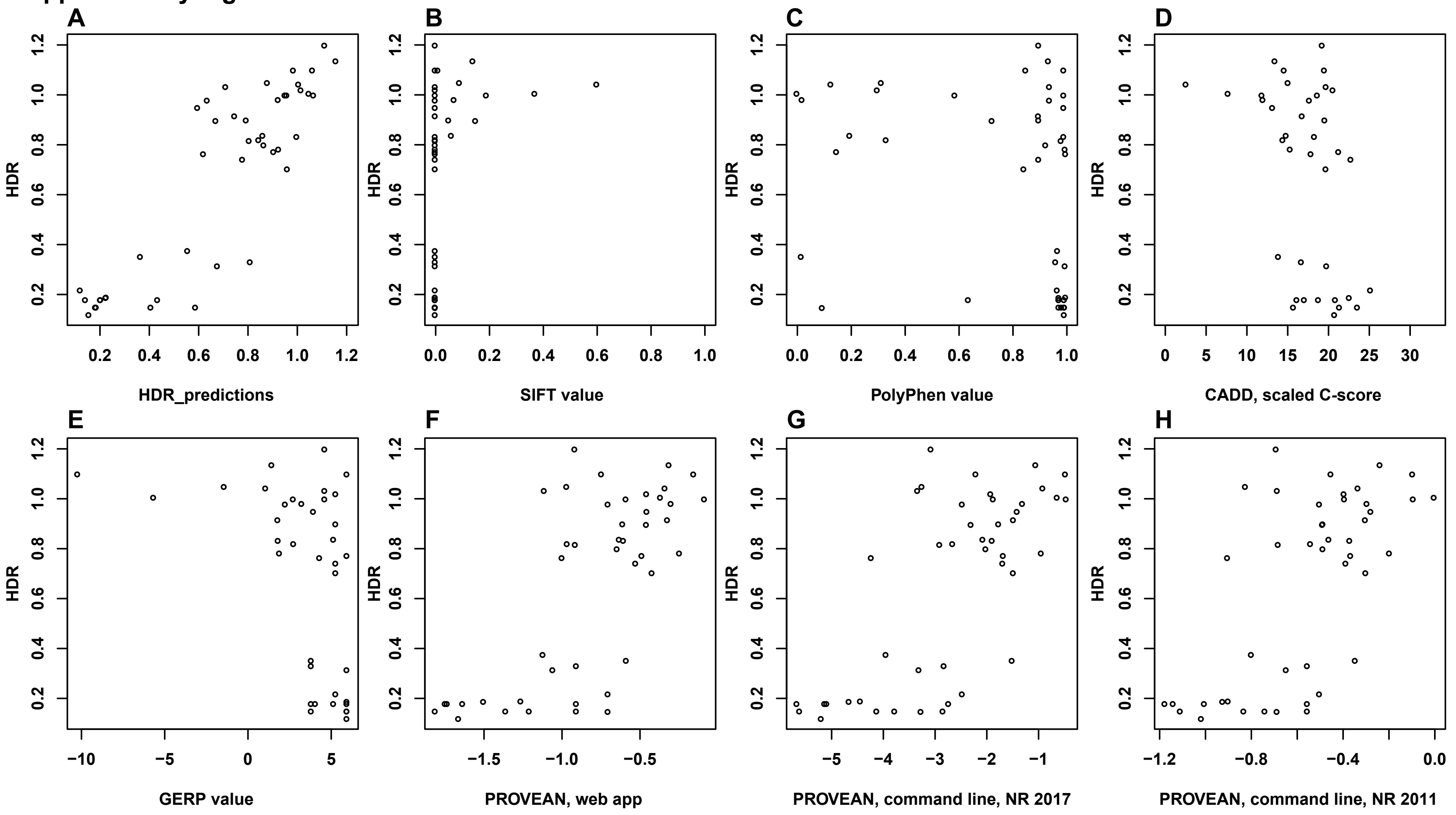

I

	Pearson		Spearman		Kendall	
	cor	p.value	Rho	p.value	Tau	p.value
HDR_predictions	+0.87	2.6E-14	+0.82	1.2E-11	+0.63	3.0E-09
Polyphen	-0.25	1.1E-01	-0.39	9.1E-03	-0.27	1.1E-02
SIFT	+0.35	2.0E-02	+0.54	1.8E-04	+0.43	3.5E-04
CADD	-0.43	7.2E-03	-0.46	4.1E-03	-0.32	5.1E-03
GERP	-0.44	6.6E-03	-0.51	1.2E-03	-0.39	1.1E-03
PROVEAN.NR2011	+0.65	1.5E-06	+0.61	1.2E-05	+0.44	3.3E-05
PROVEAN.WEB	+0.71	7.2E-08	+0.61	1.1E-05	+0.46	1.4E-05
PROVEAN.NR2017	+0.71	7.7E-08	+0.64	3.3E-06	+0.46	1.0E-05

Supplementary Figure 6

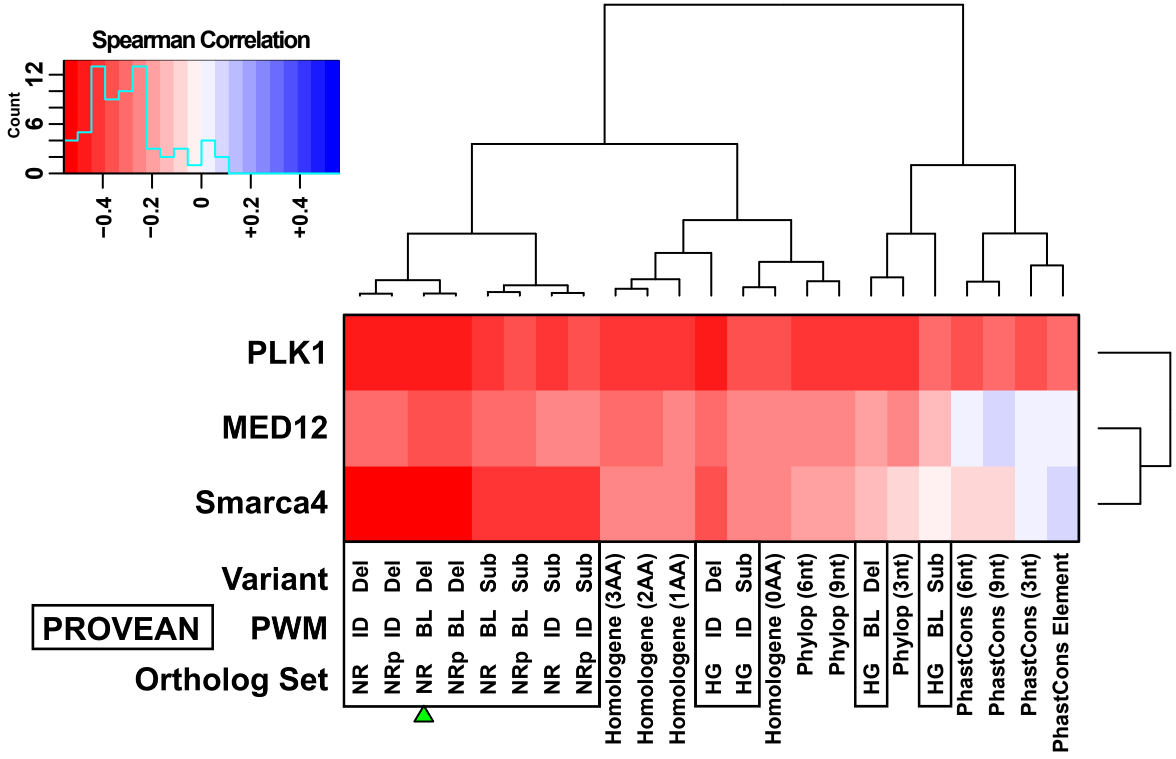

Supplementary Figure 7

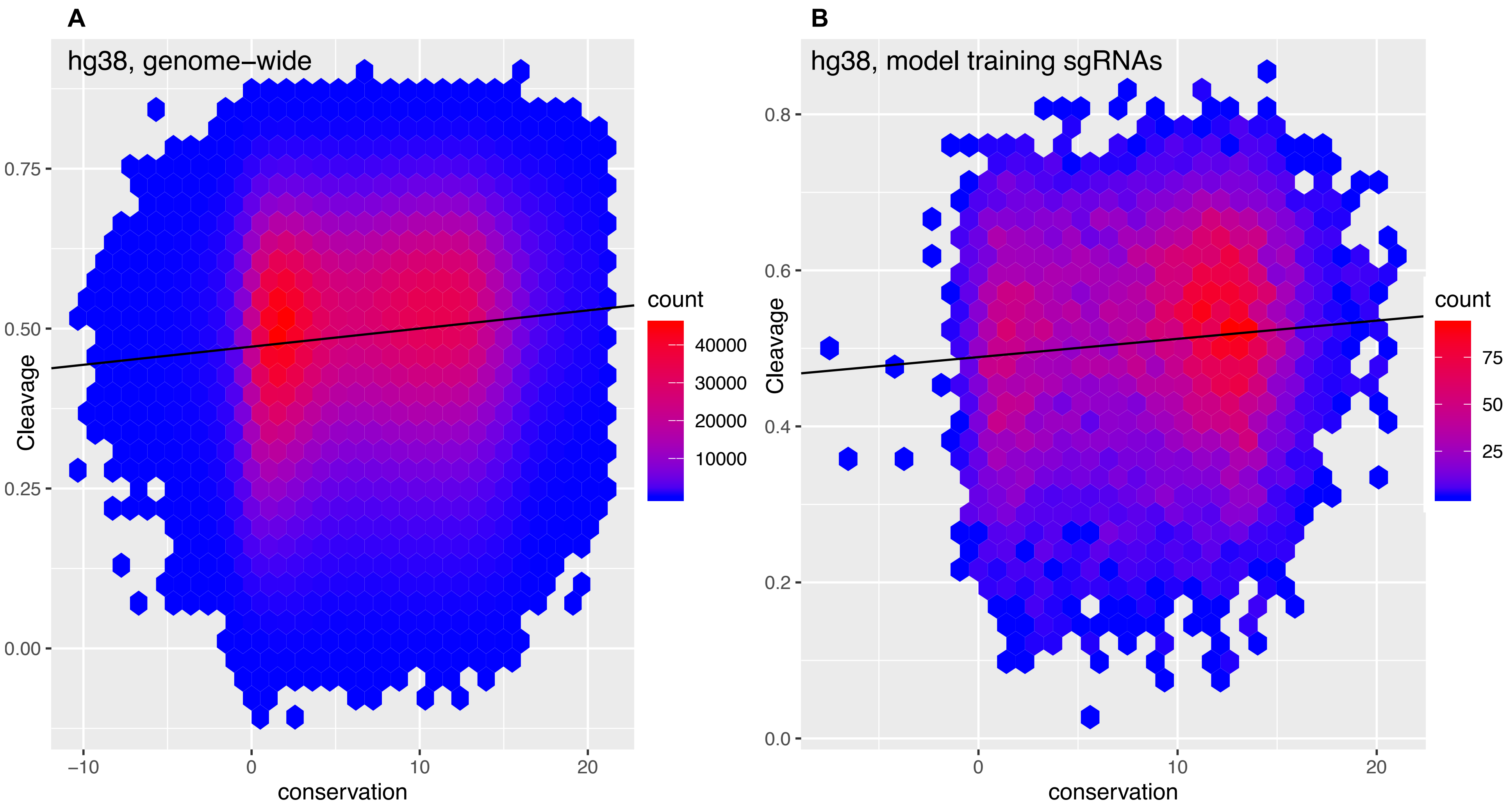

Supplementary Figure 8

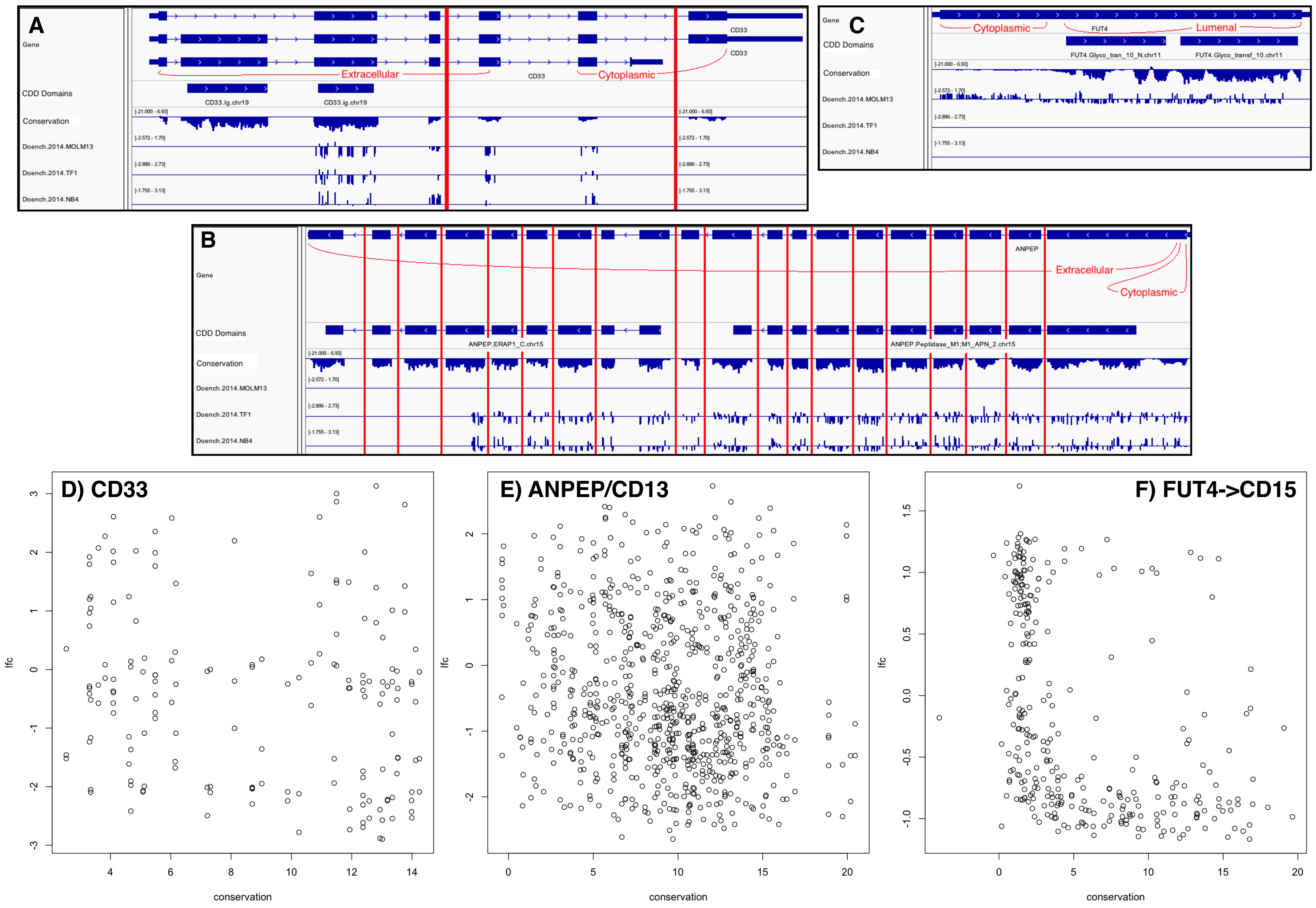

Supplementary Figure 9

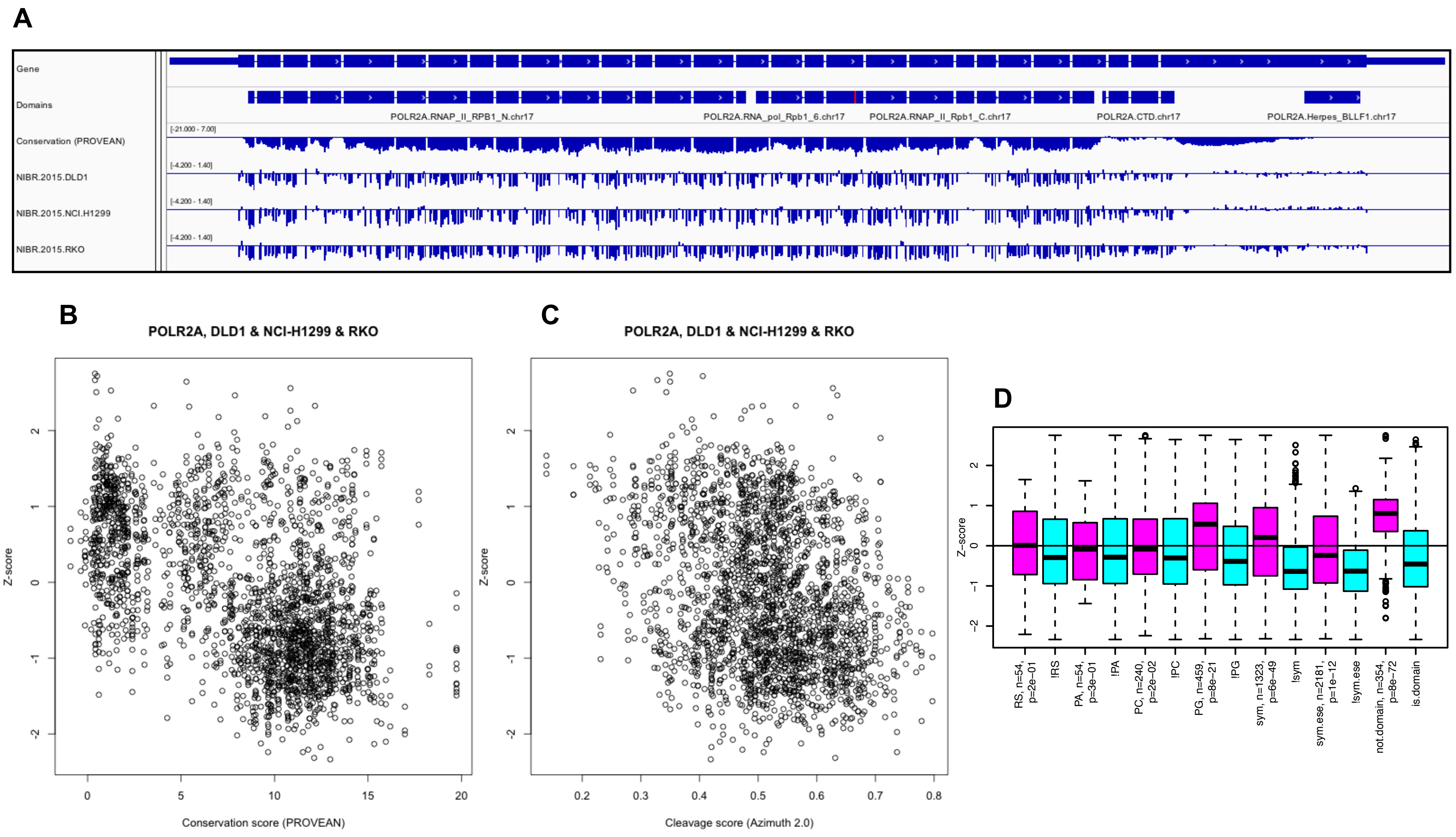

Supplementary Figure 10

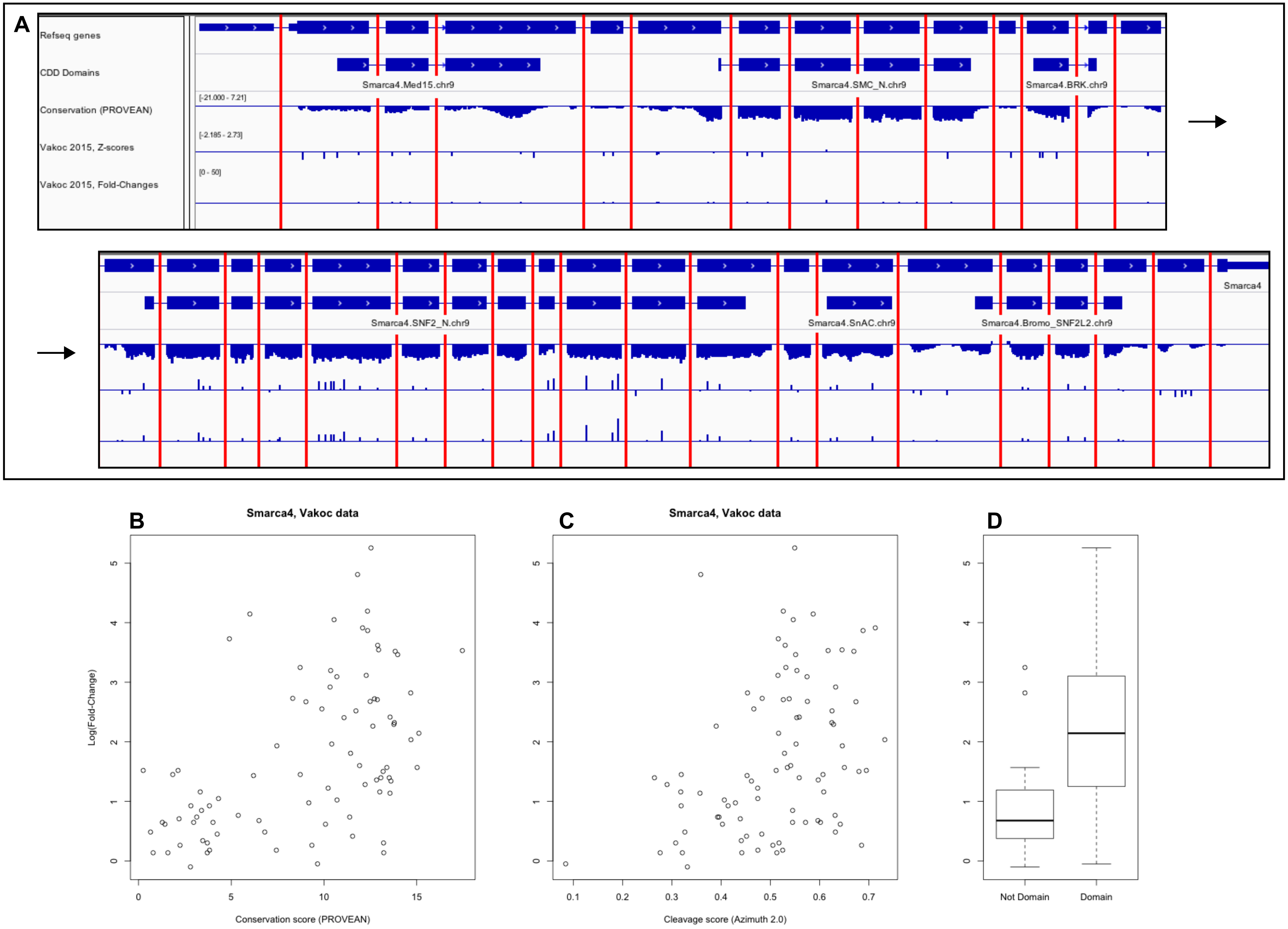

Supplementary Figure 11

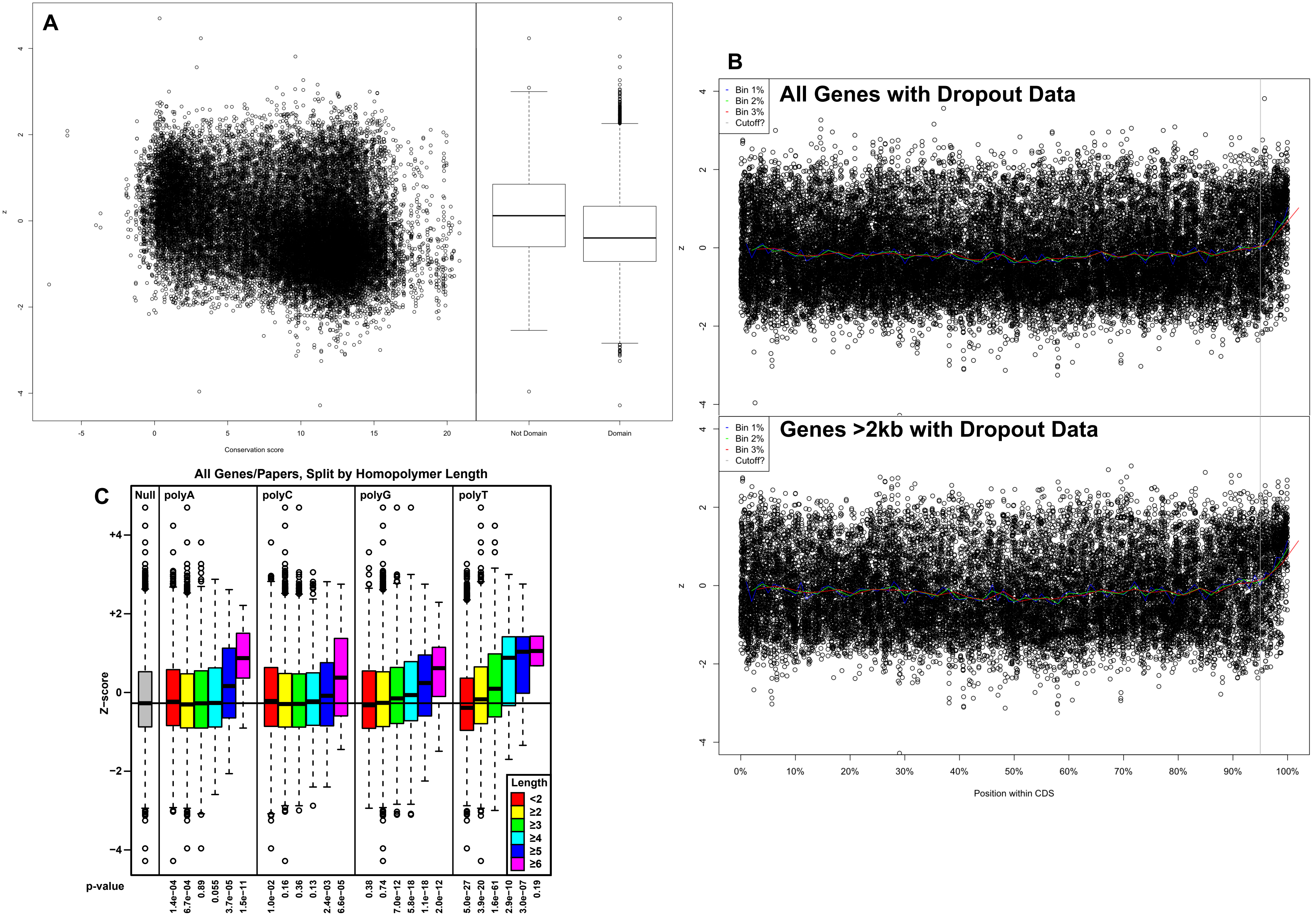

Supplementary Figure 12

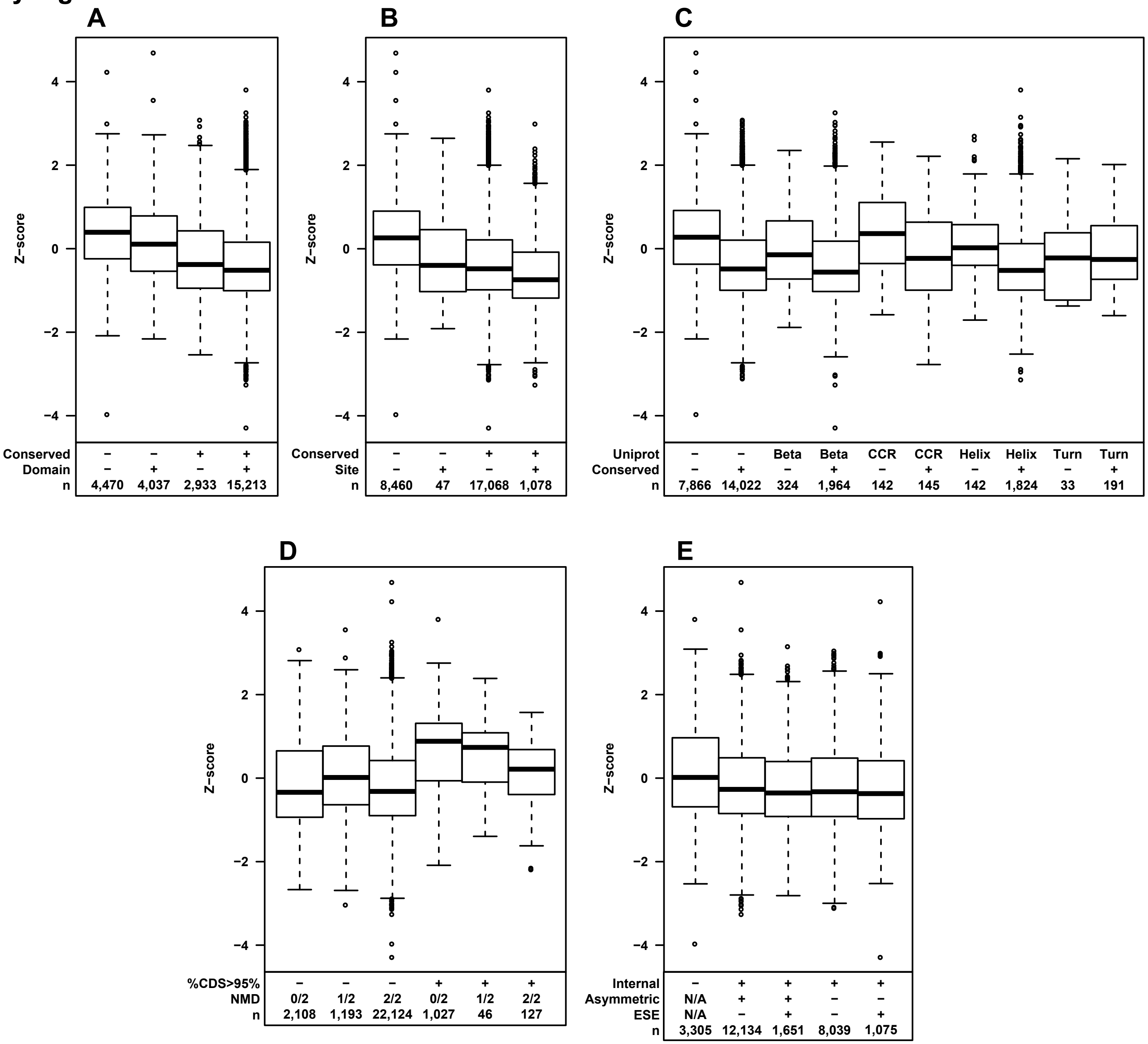

Supplementary Figure 13

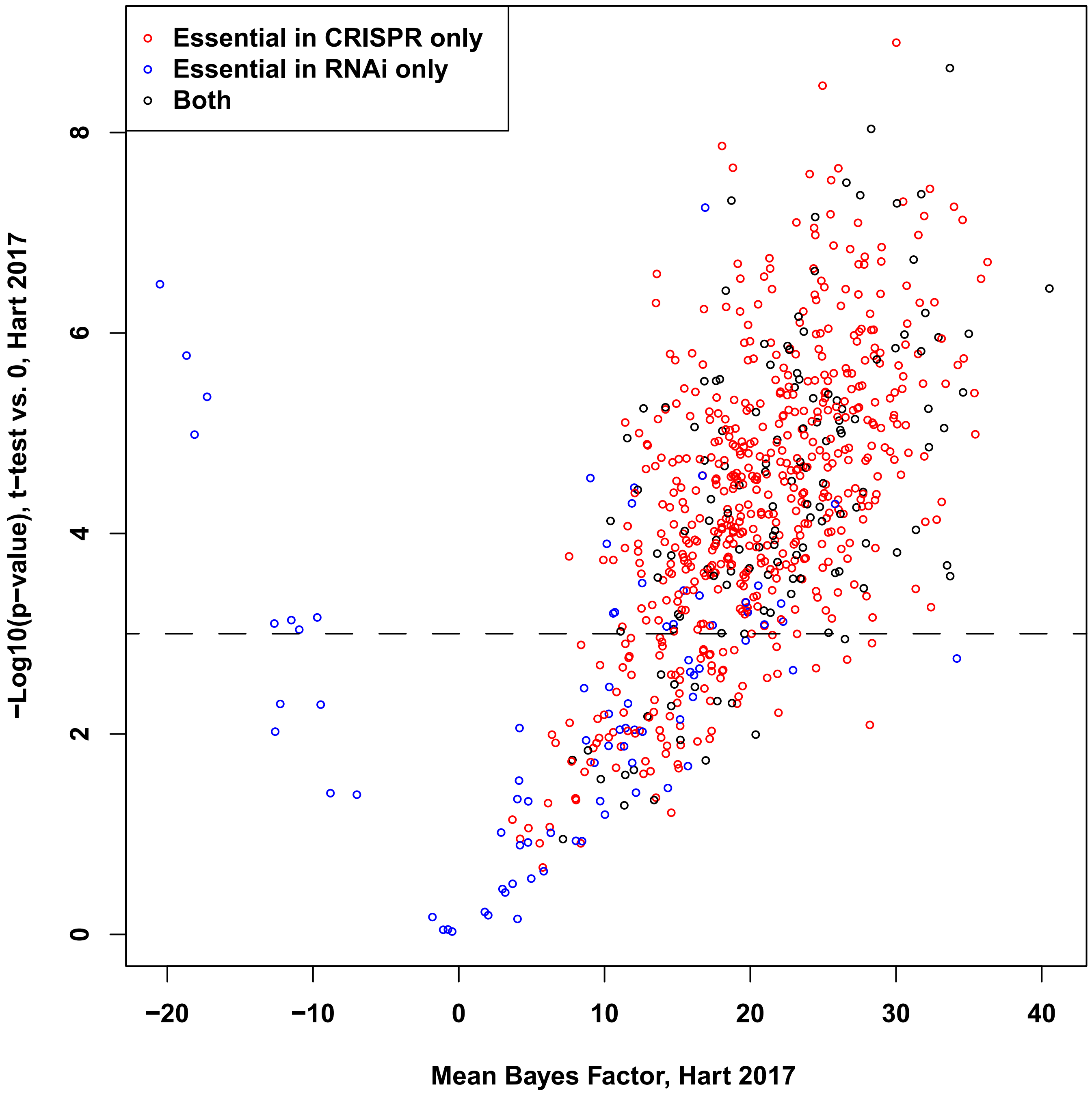

Supplementary Figure 14

A

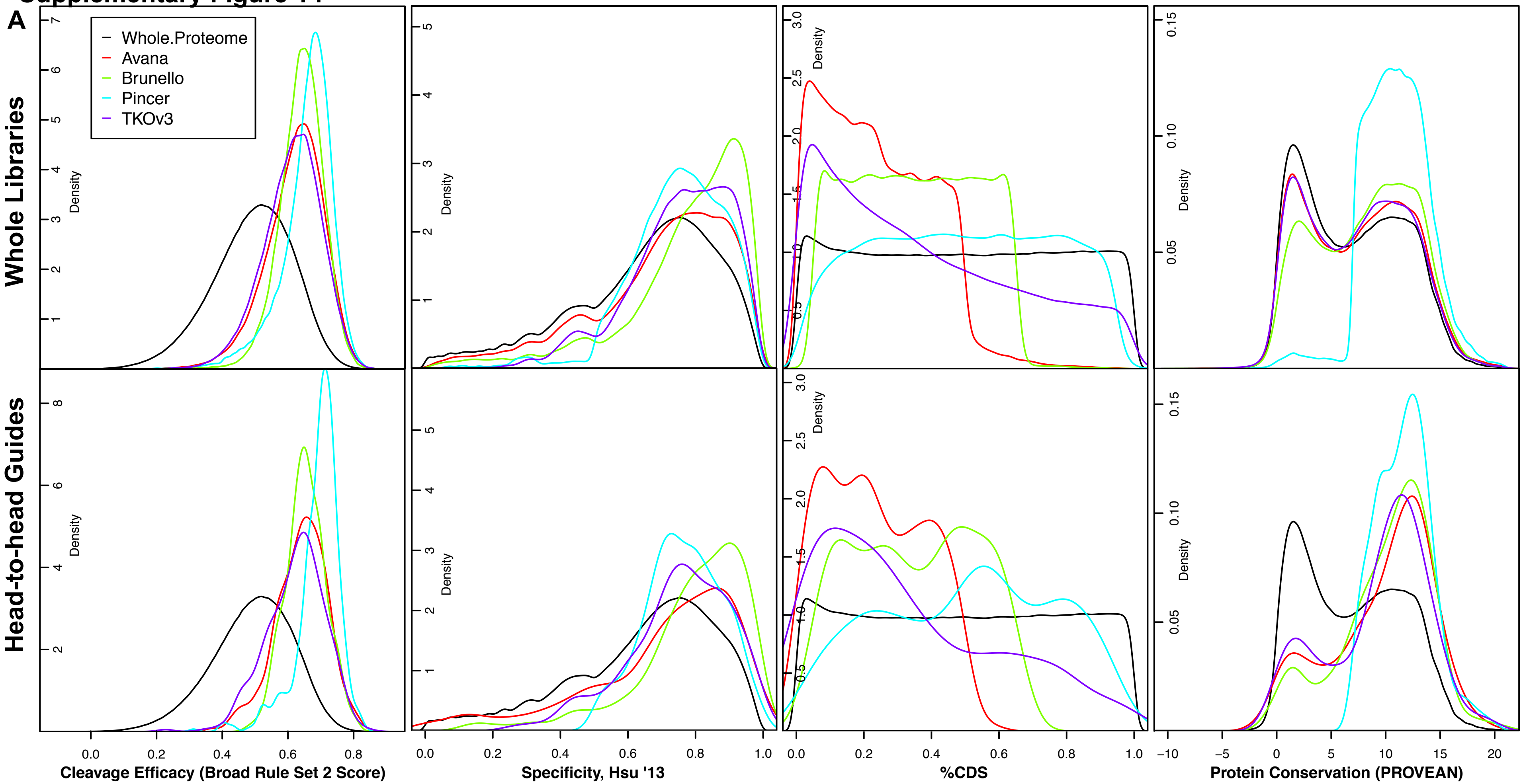

B

	Whole Libraries					Head-to-head Guides			
	Whole.Proteome	Avana	Brunello	Pincer	TKOv3	Avana	Brunello	Pincer	TKOv3
Total	5179826 (100%)	107784 (100%)	75475 (100%)	115138 (100%)	68372 (100%)	534 (100%)	360 (100%)	540 (100%)	352 (100%)
No.Esp3I	5119881 (99%)	106815 (99%)	74982 (99%)	115114 (100%)	67972 (99%)	531 (99%)	359 (100%)	540 (100%)	350 (99%)
No.SNPs	5002588 (97%)	104026 (97%)	72493 (96%)	114762 (100%)	68153 (100%)	507 (95%)	342 (95%)	540 (100%)	350 (99%)
Domain.CDD	2676175 (52%)	62000 (58%)	49294 (65%)	105296 (91%)	40005 (59%)	386 (72%)	292 (81%)	535 (99%)	265 (75%)
Domain.Pfam	2201858 (43%)	54089 (50%)	43044 (57%)	90036 (78%)	34636 (51%)	356 (67%)	255 (71%)	465 (86%)	241 (68%)
Conserved.Residue	2432487 (47%)	57844 (54%)	45593 (60%)	110663 (96%)	36468 (53%)	395 (74%)	285 (79%)	540 (100%)	255 (72%)
All.Good.Stats	945179 (18%)	30452 (28%)	28082 (37%)	100561 (87%)	24212 (35%)	219 (41%)	199 (55%)	531 (98%)	182 (52%)

Supplementary Figure 15

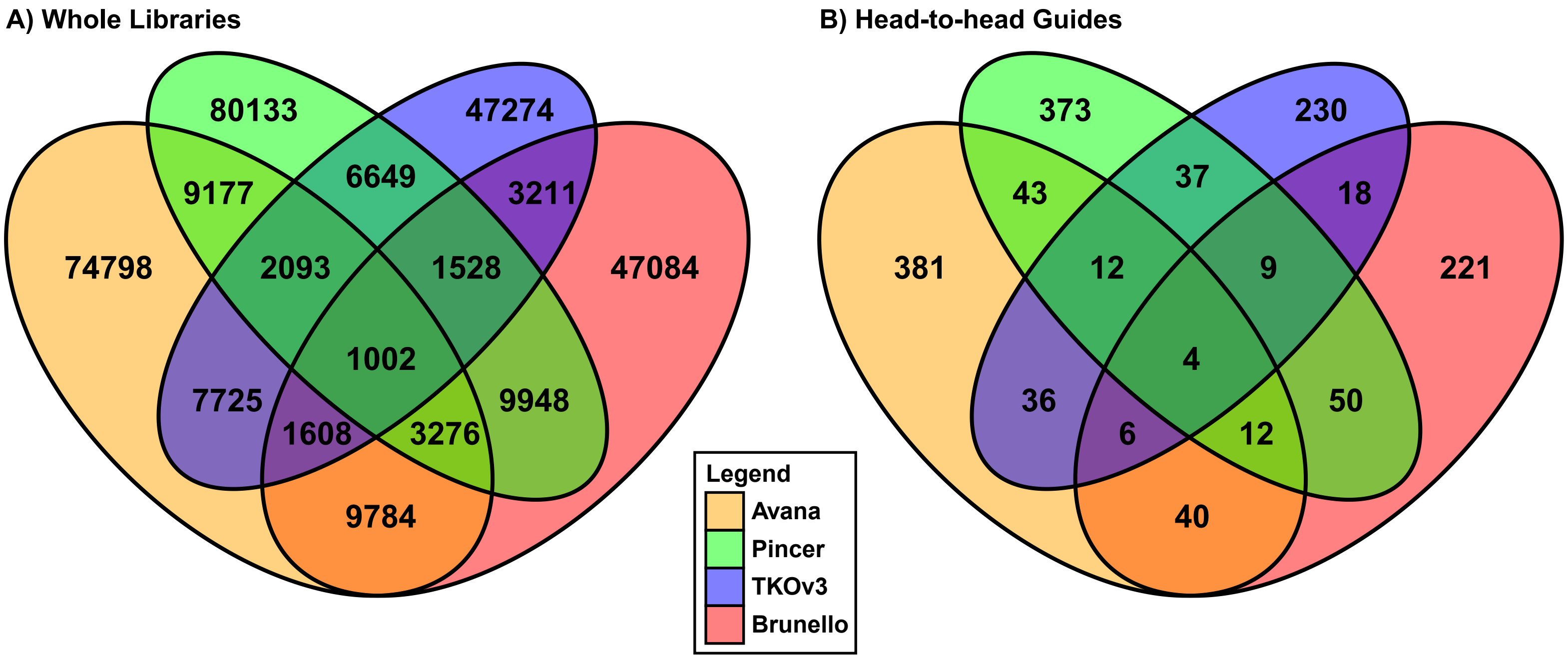

Supplementary Figure 16

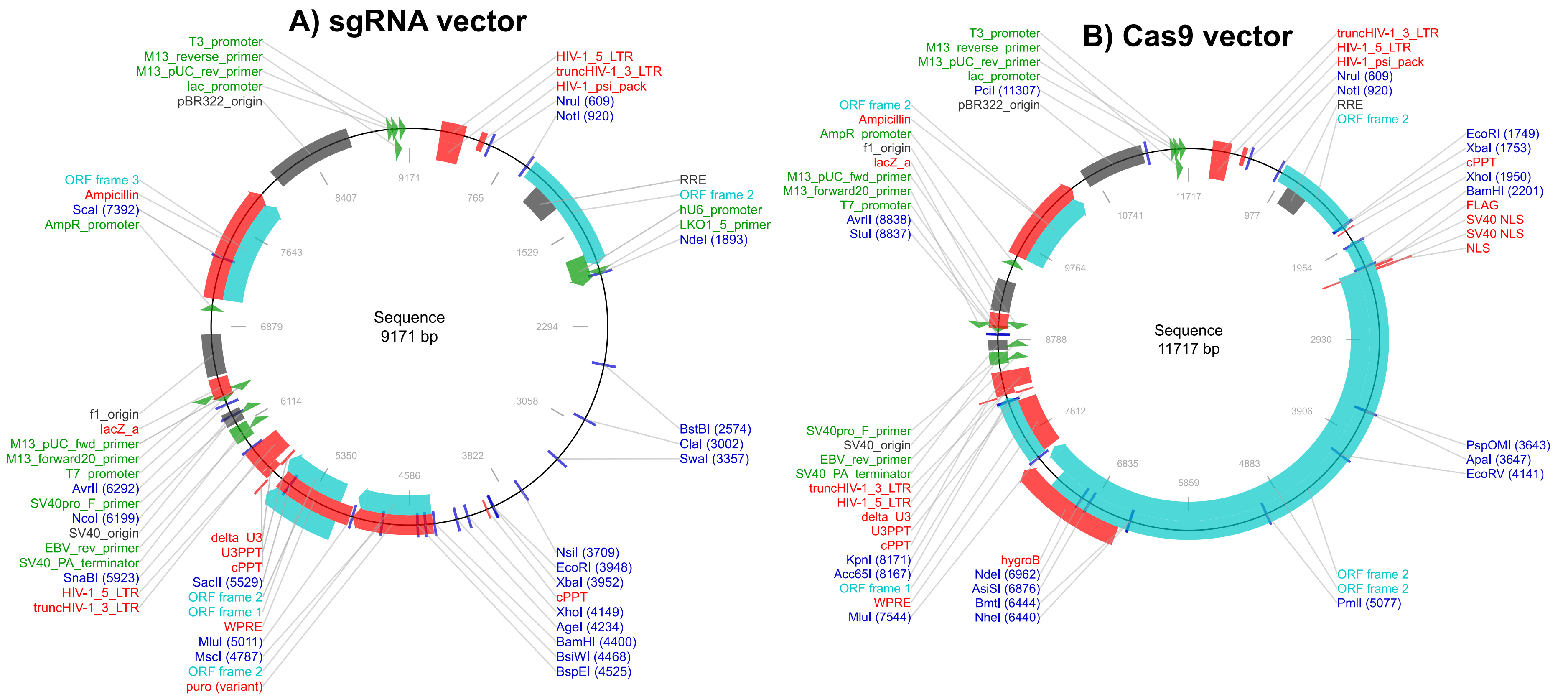

Supplementary Figure 17

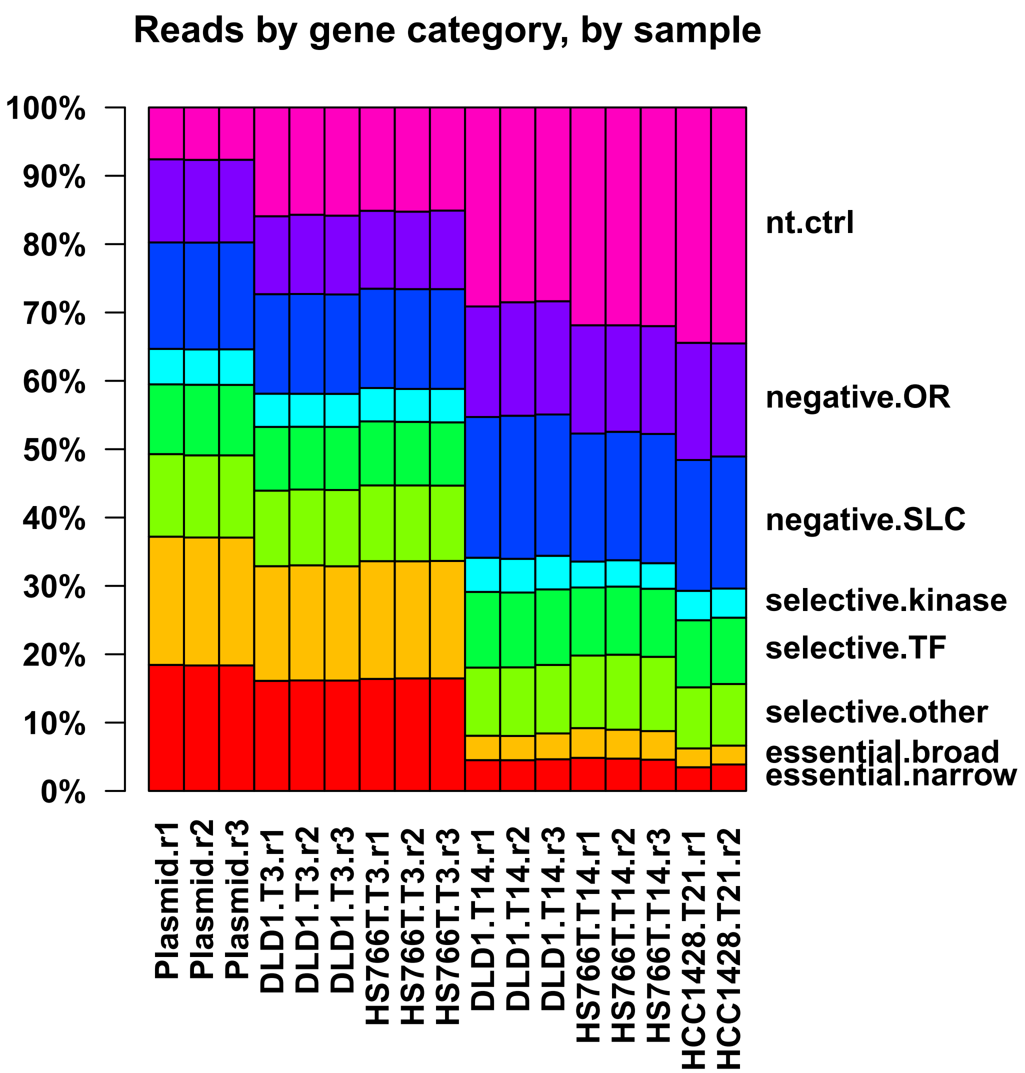

Supplementary Figure 18

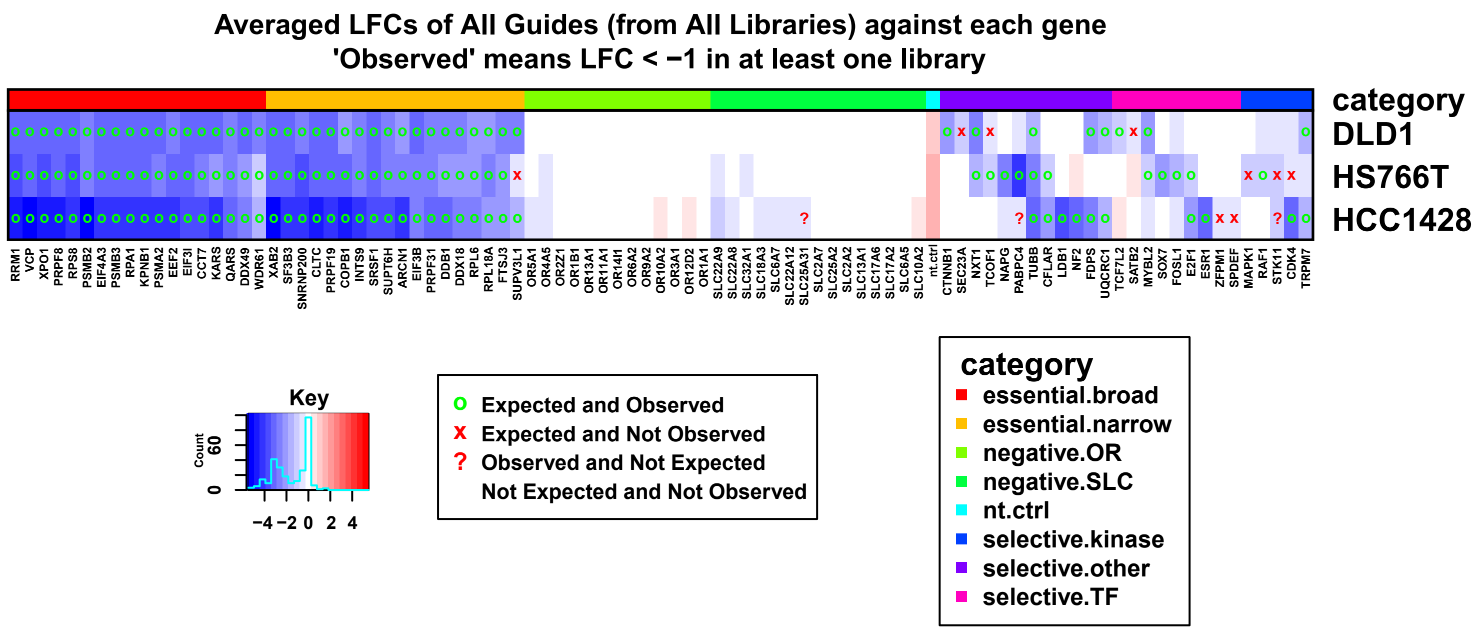

Supplementary Figure 19

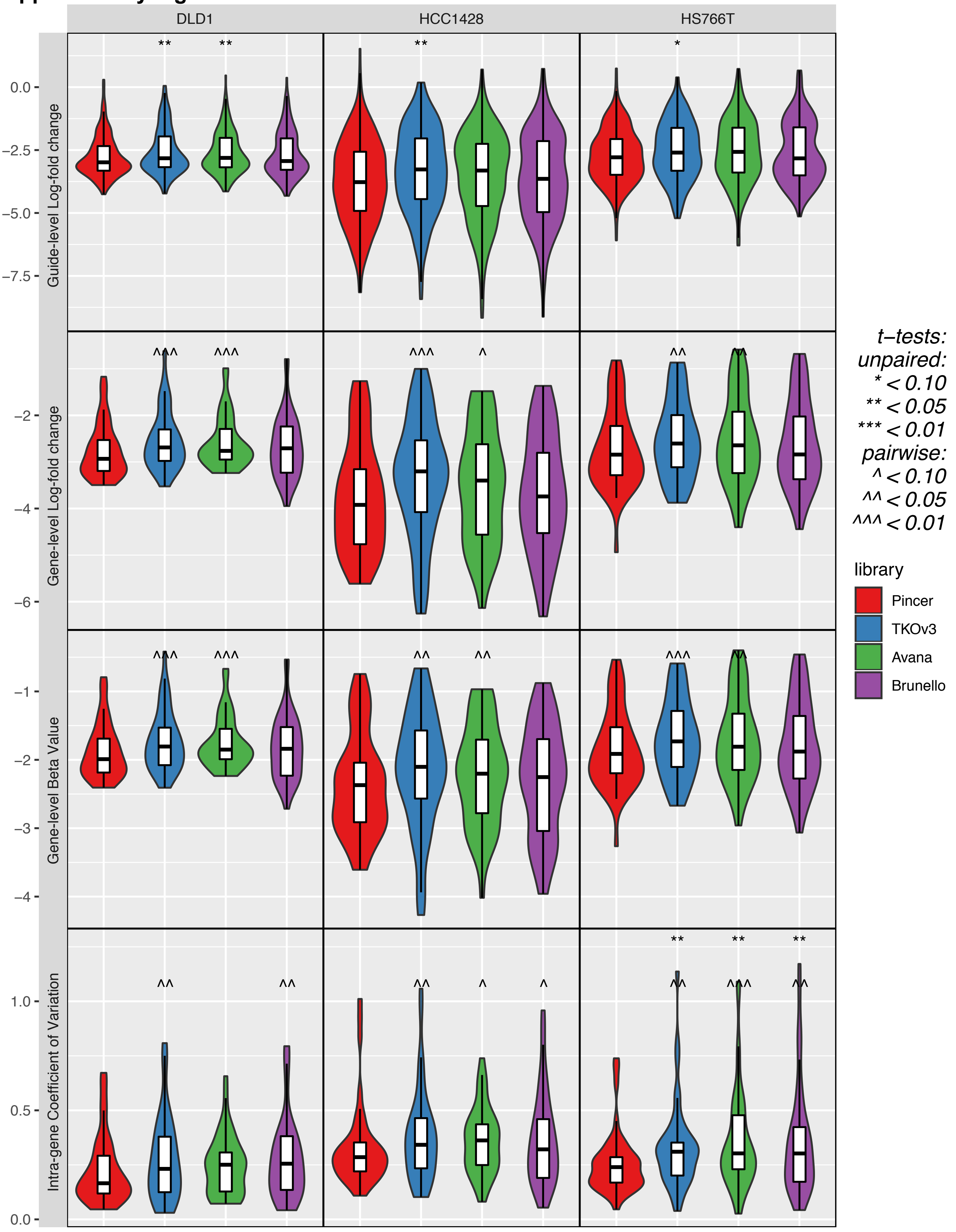

Supplementary Figure 20

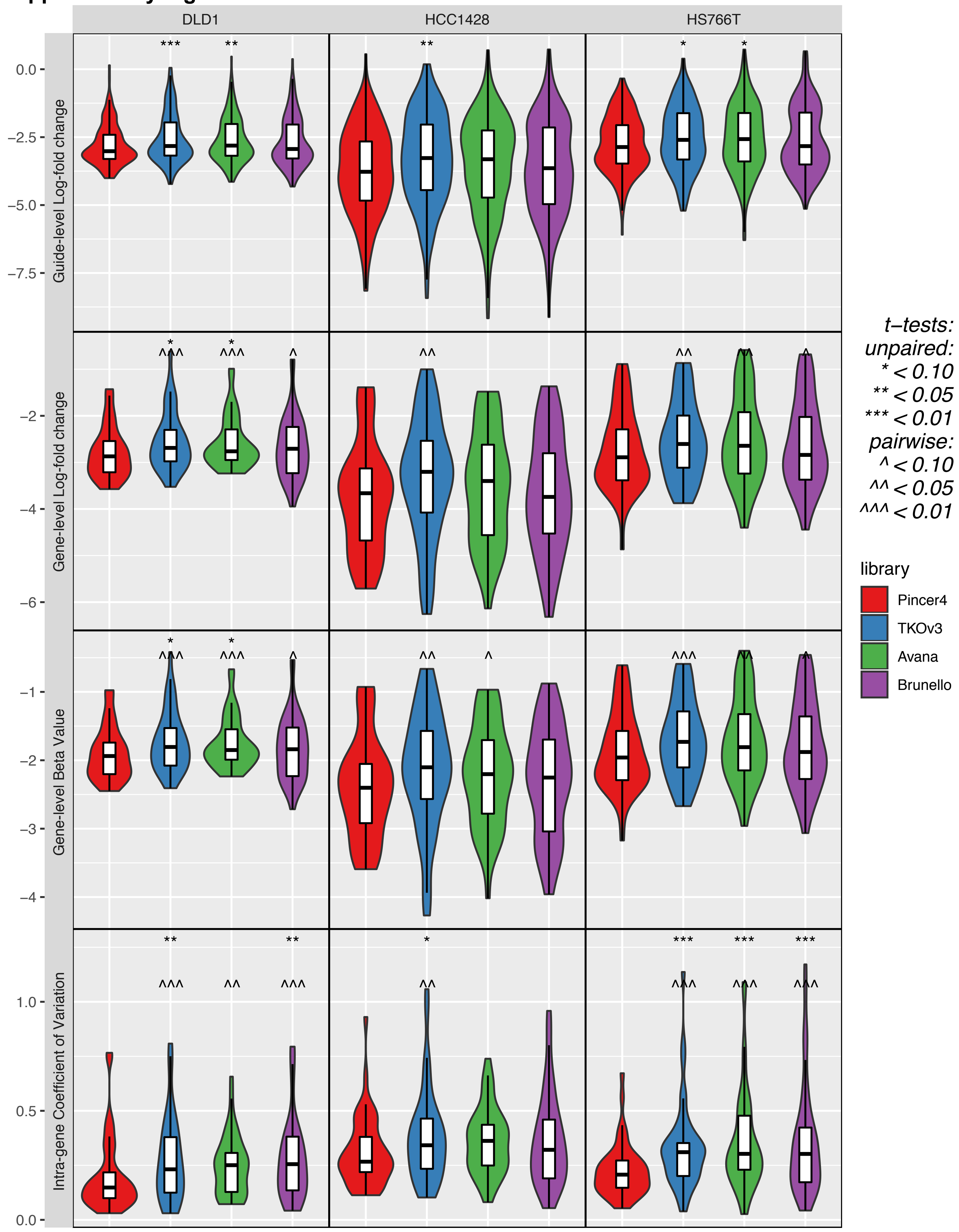

Supplementary Figure 21

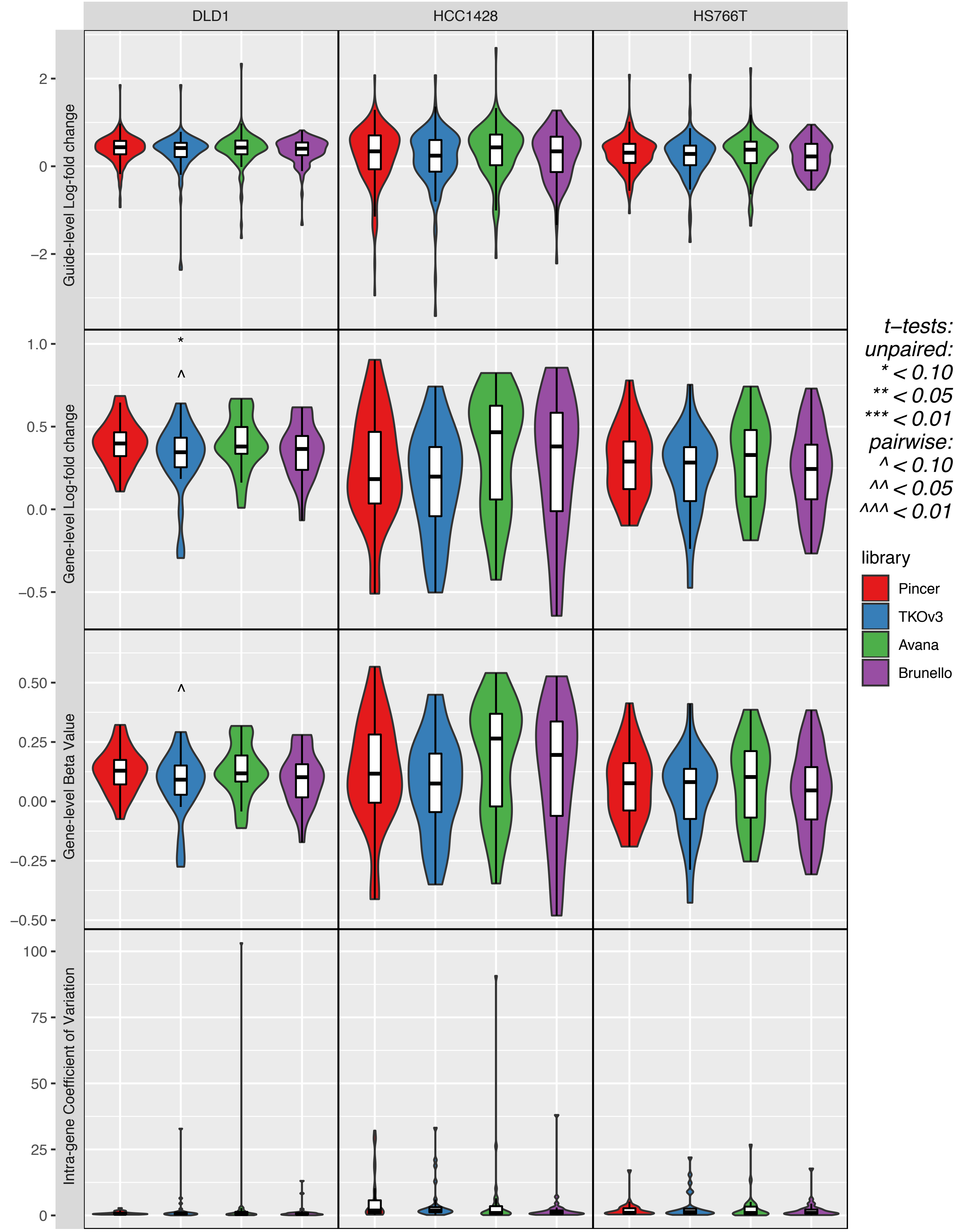

Supplementary Figure 22

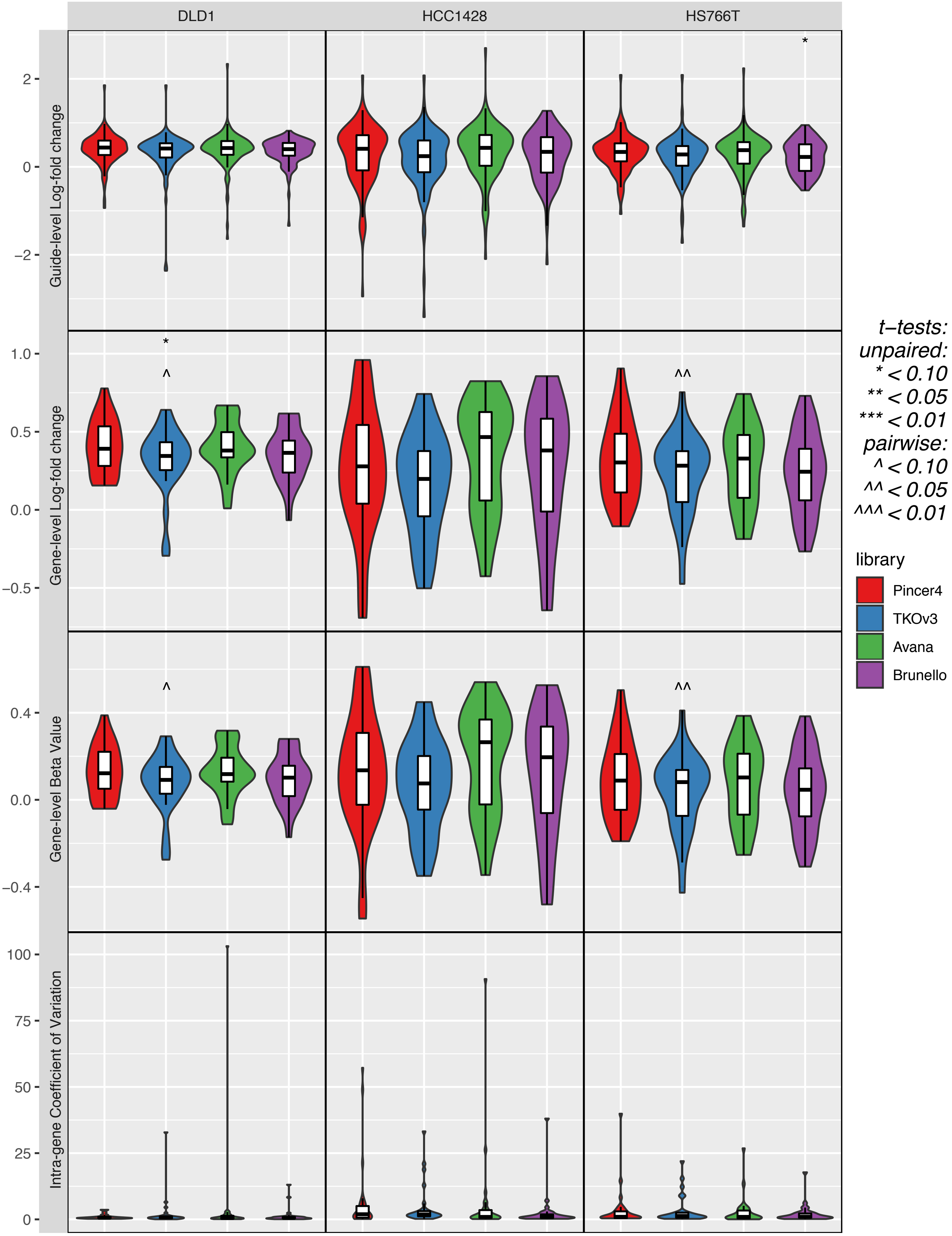

Supplementary Figure 23

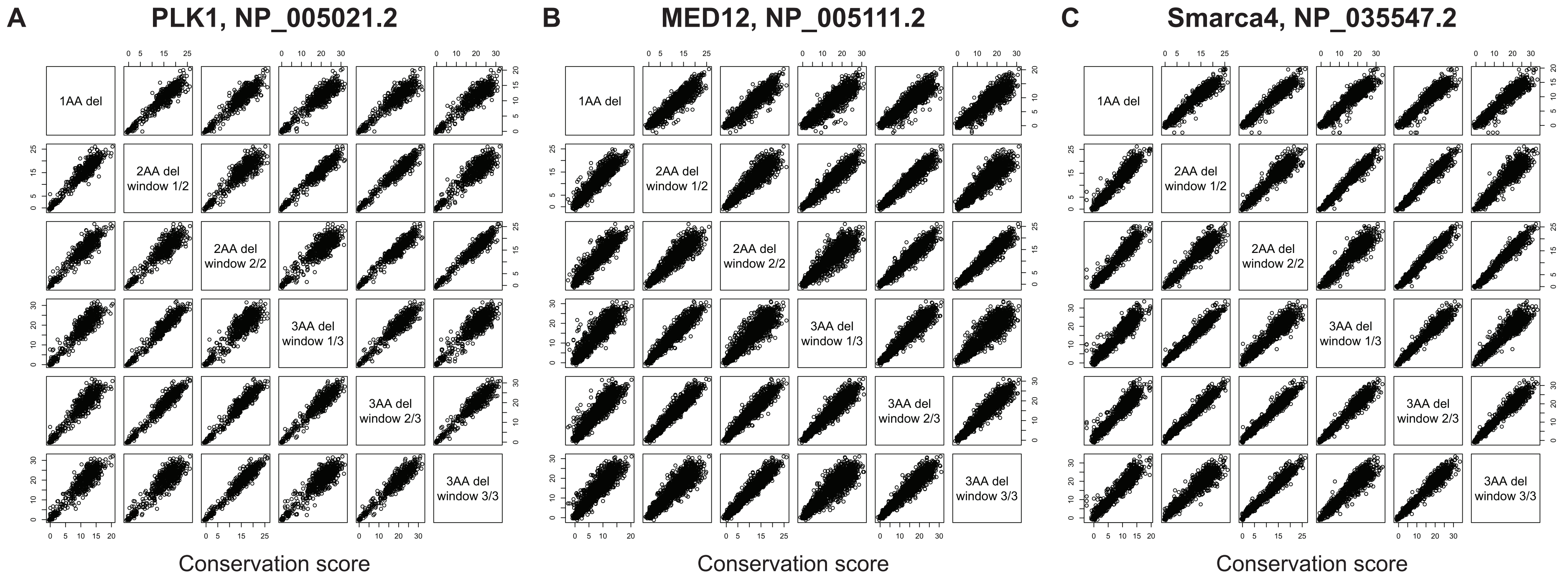

Supplementary Figure 24

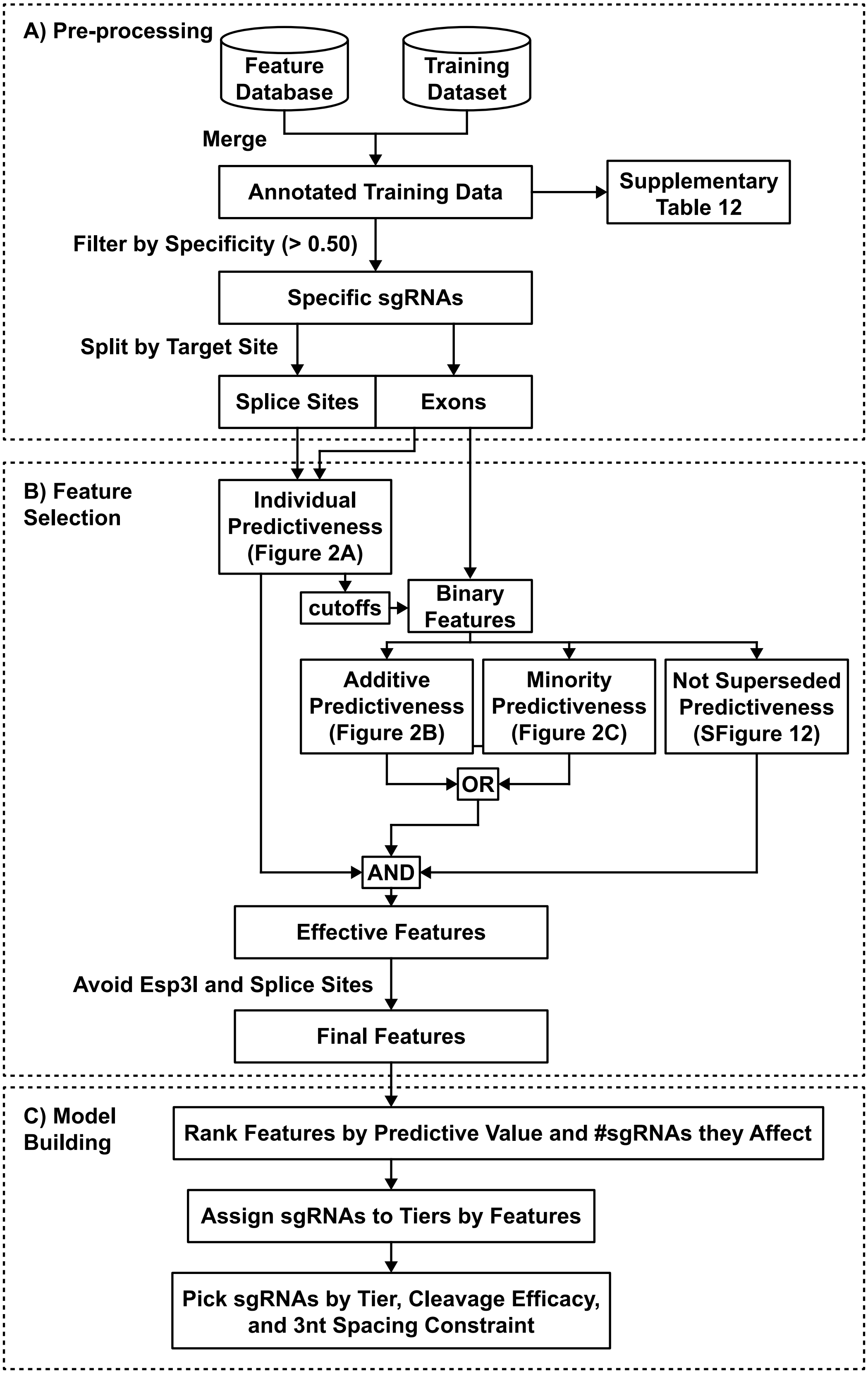

Supplementary Figure 25

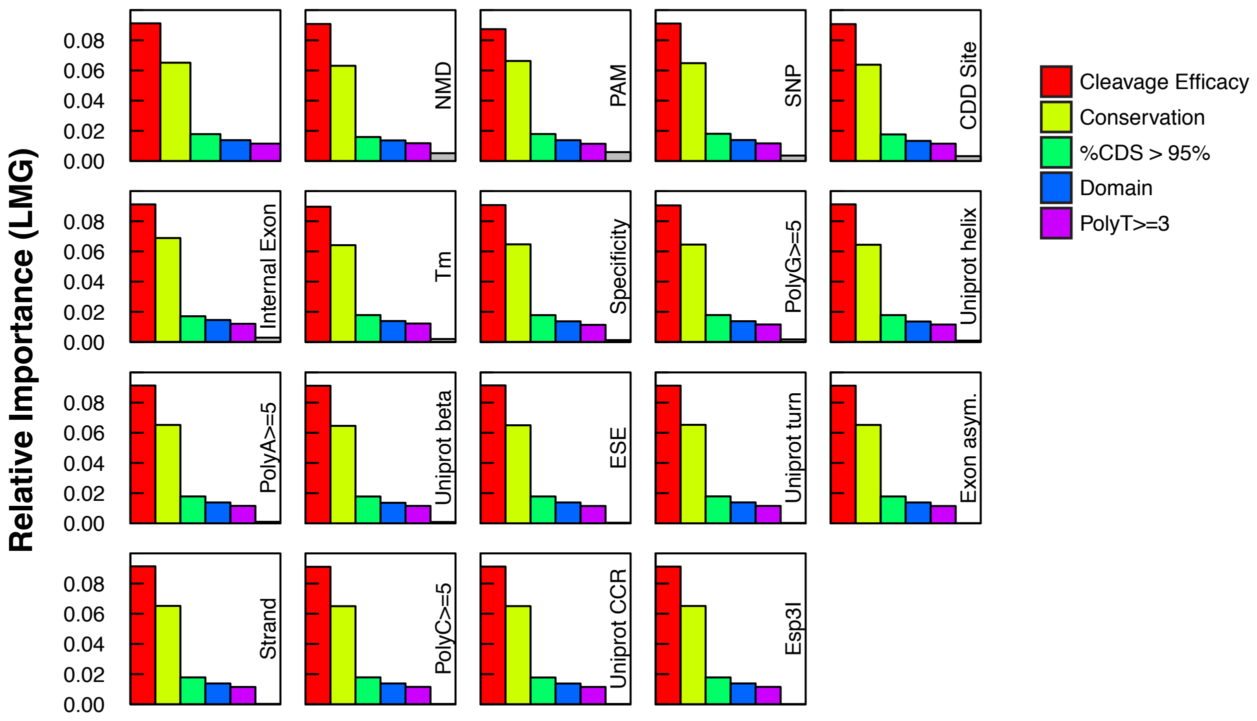

Supplement: gkaa645_Supplemental_Files [file gkaa645_supplemental_files.zip › manuscript.figures.supplemental.pdf]
